# The unique interplay between copper and zinc during catalytic carbon dioxide hydrogenation to methanol

**DOI:** 10.1038/s41467-020-16342-1

**Published:** 2020-05-15

**Authors:** Maxim Zabilskiy, Vitaly L. Sushkevich, Dennis Palagin, Mark A. Newton, Frank Krumeich, Jeroen A. van Bokhoven

**Affiliations:** 1 Laboratory for Catalysis and Sustainable Chemistry, Paul Scherrer Institute, 5232 Villigen, Switzerland; 2 Institute for Chemistry and Bioengineering, ETH Zurich, Vladimir-Prelog-Weg 1, 8093 Zürich, Switzerland

**Keywords:** Catalytic mechanisms, Heterogeneous catalysis, Reaction kinetics and dynamics

## Abstract

In spite of numerous works in the field of chemical valorization of carbon dioxide into methanol, the nature of high activity of Cu/ZnO catalysts, including the reaction mechanism and the structure of the catalyst active site, remains the subject of intensive debate. By using high-pressure operando techniques: steady-state isotope transient kinetic analysis coupled with infrared spectroscopy, together with time-resolved X-ray absorption spectroscopy and X-ray powder diffraction, and supported by electron microscopy and theoretical modeling, we present direct evidence that zinc formate is the principal observable reactive intermediate, which in the presence of hydrogen converts into methanol. Our results indicate that the copper–zinc alloy undergoes oxidation under reaction conditions into zinc formate, zinc oxide and metallic copper. The intimate contact between zinc and copper phases facilitates zinc formate formation and its hydrogenation by hydrogen to methanol.

## Abstract (web summary)

In spite of numerous works, the nature of high activity of Cu/ZnO catalyst in methanol synthesis remains the subject of intensive debate. Here, the authors study the carbon dioxide hydrogenation mechanism using high-pressure operando techniques which allow them to unify different, seemingly contradicting, models.

## Introduction

Carbon dioxide is a principal anthropogenic pollutant, rising level of which has a detrimental effect on the environment of the Earth. It is a greenhouse gas and a major driver for climate change, as well as ocean acidification^1–3^. Several technologies, including capture and storage or chemical conversion into value added chemicals, are actively being pursued to manage the concentration of carbon dioxide in the Earth’s atmosphere^4,5^. Chemical valorization of carbon dioxide into methanol, which can be used as a liquid fuel additive or precursor to other chemicals, such as formaldehyde, dimethyl terephthalate, acetic acid, and hydrocarbons, receives much attention due to an increased production of renewable energy and the need to decrease emissions of greenhouse gases into the environment^6–11^.

Among a variety of heterogeneous and homogeneous catalysts that can convert carbon dioxide into methanol, the Cu/ZnO/Al_2_O_3_ commercial catalyst for methanol synthesis from syngas mixture shows promising results^9,12^. Despite extensive efforts to improve catalyst activity^13–22^, conversion of carbon dioxide to methanol continues to be an exciting problem in chemical engineering. In particular, the unambiguous understanding of the reaction mechanism and the structure of active sites of the catalyst is essential to achieve high methanol yield and selectivity.

Multiple research groups contributed to this topic over the past decades, suggesting different active species involved in the methanol synthesis reaction. The most commonly discussed sites for carbon dioxide hydrogenation in a syngas mixture are (1) a surface copper–zinc alloy formed via partial reduction of zinc oxide, (2) metallic copper steps decorated with zinc atoms, and (3) oxygen vacancies active due to a junction effect^13,14,23–25^. Kattel et al.^15^. and Großmann et al.^26^ ascribed the active sites to the copper–zinc oxide interface, which triggered a debate about the role of zinc oxide^27,28^. Assessment of these possible sites was made using multiple physico-chemical techniques, including X-ray photoemission spectroscopy, transmission electron microscopy (TEM), temperature-programmed reaction (TPR), and probe molecule adsorption, supported by density functional theory calculations and kinetic Monte Carlo simulations. However, utilization of these techniques is associated with natural limitations to the applied measurement conditions, most notably the pressure. Most previous studies, therefore, have been based on experiments under conditions (typically, vacuum, low temperature) that are far away from the real catalytic experiment (>15 bar; 513–553 K). Hence, even a simple comparison and systematization of the existing experimental data is a rather complicated challenge.

This motivated us to address this challenge by performing operando characterization of the catalyst and the process under relevant catalytic conditions of carbon dioxide hydrogenation. Based on the results of steady-state isotope transient kinetic analysis coupled with infrared spectroscopy (SSITKA-FTIR) together with time-resolved X-ray absorption spectroscopy (XAS) and X-ray diffraction (XRD), we present the reaction intermediates, the pathways of their formation, and transformations of the structure of the copper–zinc catalyst that accompany differing treatments that result in the formation of the active catalyst. The commonly discussed copper–zinc alloy, which forms under highly reducing conditions, under CO_2_/H_2_ atmosphere undergoes transformation into a mixture of zinc oxide and formate. Our results reveal that formate associated with zinc is the main reactive intermediate responsible for the formation of methanol from carbon dioxide. This study considerably augments our understanding of the reaction mechanism of carbon dioxide hydrogenation over copper–zinc catalyst and identifies the unique interplay between copper and zinc in the Cu/ZnO system, serving as a guideline for the further rational design of a new class of catalyst for this highly important and industrially relevant reaction.

## Results

### SSITKA-FTIR mechanistic study

**Fig. 1:**
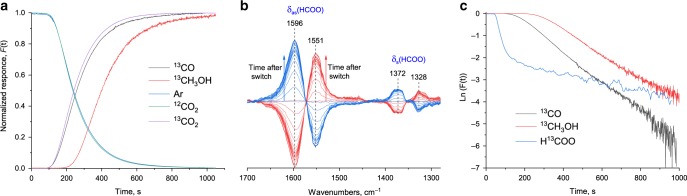
Results of SSITKA-FTIR experiment. **a** Normalized isotopic transient response curves following the switch from ^12^CO_2_/H_2_ to ^13^CO_2_/H_2_ mixture with a total flow of 25 cm^3^ min^−1^, H_2_/CO_2_ molar ratio of 3 at 533 K and 15 bar during the reaction over CZA catalyst. **b** Time-resolved in situ FTIR difference spectra of surface species formed during switch from ^12^CO_2_/H_2_ to ^13^CO_2_/H_2_ (red spectra) and ^13^CO_2_/H_2_ to ^12^CO_2_/H_2_ (blue spectra) mixture at 533 K and 15 bar during the reaction over CZA catalyst. **c** Comparison of logarithmic transient responses of methanol, carbon monoxide and formate species from SSITKA-FTIR over CZA.

We have used a standard copper–zinc–alumina (CZA) catalyst purchased from Alfa Aesar for the methanol synthesis from a mixture of hydrogen and carbon dioxide with a molar ratio of three. Reactivity tests were performed in a typical continuous flow fixed-bed reactor with reaction products analyzed online using gas chromatography. The main reaction products were methanol and carbon monoxide with a selectivity to methanol of 43–55 mol%, depending on pretreatment conditions (Table 1). This range of selectivity allows studying the formation pathways of both methanol and carbon monoxide. The kinetic behavior of the reaction products and intermediates was accessed using SSITKA-FTIR performed at 15 bar total pressure^29^. After achieving the steady-state operation, an isotope switch from a mixture of ^12^CO_2_/H_2_ to that of ^13^CO_2_/H_2_ was made, and the responses of the main reaction products and intermediates were followed by mass spectrometry and infrared spectroscopy (Fig. 1a, b). The normalized response of ^12^CO_2_ precisely follows the signal of the inert tracer (argon), which was added for the setup holdup time correction. This behavior is associated with the fast and irreversible transformation of carbon dioxide surface species without the formation of stable intermediates^30^. In agreement, no carbonates and bicarbonates were observed in the infrared spectra (Supplementary Fig. 1)^31–34^. The carbon monoxide normalized response shows a similar trend to that of carbon dioxide, featuring a slight delay that indicates the presence of intermediate surface species. In contrast, the signal for the ^13^C-labeled methanol appears with considerably longer delay, indicating a stepwise conversion of carbon dioxide into methanol along with possible re-adsorption of the latter over the surface of the catalyst.

**Table 1:** Results of PCA analysis of Zn K-edge XANES as well as methanol selectivity and productivity during carbon dioxide hydrogenation experiment for CZA catalyst under different pretreatment conditions (measured at 533 K, 15 bar and H_2_/CO_2_ molar ratio of 3, carbon dioxide conversion ≈2%).

Pretreatment conditions	Methanol production, mmol g^−1^ min^−1^	Methanol selectivity, %	Reduced zinc, mol%
673 K and 15 bar in H_2_	0.38 ± 0.02	54 ± 2	49 ± 1
533 K and 15 bar in H_2_	0.44 ± 0.02	43 ± 2	16 ± 1
673 K and 15 bar in H_2_; after that 15 bar in CO_2_/H_2_ mixture at 573 K	0.28 ± 0.02	48 ± 2	33 ± 1

To access the nature of the intermediate species, infrared spectra were acquired during the ^12^CO_2_/H_2_ to ^13^CO_2_/H_2_ switches (Supplementary Fig. 1). Figure 1b represents difference spectra obtained during these switches. The positions of the bands at 1596 and 1372 cm^−1^ correspond to the asymmetric and symmetric vibrations of the formate species formed in the ^12^CO_2_/H_2_ reaction mixture^31–34^. Importantly, no bands due to methoxy species were detected. This can be attributed to the formation of water during the reaction, which may facilitate desorption of methanol. The switch to the ^13^C-labeled reacting mixture leads to an immediate decrease of the intensity of the bands due to the unlabeled formate, with simultaneous appearance of new bands at 1551 and 1328 cm^−1^ assigned to the ^13^C-labeled surface formate. The total conversion of the unlabeled formate into the labeled one is completed in about 20 min; the switch from ^13^CO_2_/H_2_ back to ^12^CO_2_/H_2_ shows the full reversibility of the isotope exchange, hence excluding the formation of unreactive spectator-type formate species leading to the formation of methanol.

Figure 1c shows the logarithmic transient responses of methanol and carbon monoxide and indicates a faster rate of formation of carbon monoxide compared with methanol. In contrast, the transient response of formate species reveals the presence of two distinct processes characterized by different reaction rates. The isotope exchange of formate immediately upon the isotope switch (50–100 s) is much faster than that after some time (100–1000 s), which is indicative of the presence of two classes of formates with distinctively different reactivity, probably associated with different localization with respect to copper^35^.

### Operando XAS study

FTIR alone, however, does not reveal the location of the formate species on the bifunctional copper–zinc catalyst. Therefore, operando time-resolved Cu and Zn *K*-edge XAS was used to probe the active sites of the catalyst. The activation and reaction protocols were identical to those described for catalytic tests and the SSITKA-FTIR experiment. XAS enables monitoring the perturbation of the oxidation state and the local environment of the catalyst, induced by switching between the CO_2_/H_2_ gas mixture and either pure hydrogen or carbon dioxide at 15 bar total pressure.

The normalized Zn K-edge X-ray absorption near edge structure (XANES) spectrum of the CZA catalyst pretreated in a flow of helium (Supplementary Fig. 2) shows a main peak located at 9669.2 eV with a shoulder at 9664.0 eV, which is indicative of the dominant presence of the wurtzite zinc oxide phase^36^. After the activation in hydrogen, a characteristic pre-edge shoulder at 9659 eV appeared in the Zn K-edge spectrum. The assignment of this peak to copper–zinc alloy was made based on a H_2_-TPR-XAS experiment at 15 bar, wherein almost complete transformation of zinc oxide into copper–zinc alloy was achieved at 873 K (Supplementary Figs. 2–4, Supplementary Note 1).

**Fig. 2:**
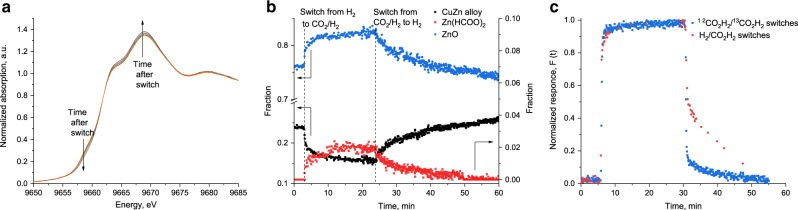
Results of operando XAS experiment. **a** Time-resolved Zn K-edge XANES spectra evolution during operando switch from pure hydrogen to CO_2_/H_2_ gas reaction mixture at 15 bar and 533 K over CZA catalyst. **b** The relative fractions of zinc oxide wurtzite, zinc formate and copper–zinc alloy determined from PCA analysis of XANES spectra as function of time after switch from hydrogen to CO_2_/H_2_ mixture and back at 533 K and 15 bar over CZA catalyst. **c** Normalized transient response curves of formate species from the FTIR experiment during ^12^CO_2_H_2_/^13^CO_2_H_2_ (blue) and H_2_/^12^CO_2_H_2_ (red) switches at 533 K and 15 bar over CZA catalyst.

After reduction in hydrogen at 533 K, the reaction mixture was switched to CO_2_/H_2_. Figure 2a shows the evolution in the Zn K-edge XANES during this switch. The pre-edge shoulder decreases as a result of oxidation of that fraction of zinc that has been incorporated into a copper–zinc alloy through reduction in hydrogen. This indicates that the zinc structure and oxidation state are a function of the reduction potential of the feedstock, and that the high temperature and high partial pressure of hydrogen promote copper–zinc alloy formation^14,37^.

Principal component analysis (PCA) reveals that wurtzite-type phase is the major cationic zinc component in this system (Supplementary Figs. 5 and 6, Supplementary Note 1). Besides the partial oxidation of the copper–zinc alloy after switching to the CO_2_/H_2_ reaction mixture, an immediate formation of zinc formate was observed. PCA quantification of the XAS spectra acquired during catalytic carbon dioxide hydrogenation indicates that, along with the wurtzite zinc oxide phase, around 16 mol% of zinc is present in a reduced form as copper–zinc bulk alloy, and around 2 mol% as surface zinc formate (Fig. 2b). Cu K-edge XANES and EXAFS (Supplementary Figs. 7–10, Supplementary Note 1) show that copper is always present in a metallic form and does not undergo oxidation to Cu^I^ or Cu^II^ species (and/or formation of copper formate) during the hydrogen to CO_2_/H_2_ switch. Copper formate species, even if they were formed, could not be hydrogenated to methanol; instead such copper formates undergo decomposition to carbon dioxide and hydrogen during transient switch to deuterium^38,39^. Equally, however, in the spectral region of the XANES fingerprints (Supplementary Figs. 8 and 9), the features typical of partial de-alloying process are visible, suggesting the formation of metallic copper from copper–zinc alloy upon treatment in CO_2_/H_2_. Moreover, oxidation of copper is not observed even during the switch from CO_2_/H_2_ mixture to pure carbon dioxide, which has stronger oxidation properties (Supplementary Fig. 11).

After switching back to a pure hydrogen atmosphere (Fig. 2b), decomposition of zinc formate species, as well as partial re-reduction of the wurtzite-like phase with the formation of copper–zinc alloy, take place. This is in good agreement with the reversibility indicated from infrared spectroscopy, where we observed decomposition of the surface formate species after the switch to hydrogen (Fig. 2c, Supplementary Fig. 12 and Supplementary Note 2). The simultaneously detected MS-response (Supplementary Figs. 13 and 14) indicates that the main products formed during decomposition of the surface formate species under a hydrogen atmosphere are methanol and carbon monoxide. This suggests that the zinc formate species is the principal intermediate that yields methanol during carbon dioxide hydrogenation. Comparing the kinetics of the zinc formate decomposition determined by time-resolved XAS with those of the FTIR experiments and SSITKA, we can clearly see the difference upon switch to pure hydrogen. Thus, the formation of formate species or ^13^C-exchange proceed rapidly (Fig. 2b, c), while the switch from CO_2_/H_2_ mixture to hydrogen results in a much slower decomposition of formate together with the formation of copper–zinc alloy. Equally, we should keep in mind, that by switching from CO_2_/H_2_ to H_2_ and vice versa we operate at non steady-state regime, which might cause the changes in the structure of the catalyst, by exposing it to the different environment (Supplementary Figs. 15 and 16, Supplementary Note 3). In this respect, the comparison with steady state is crucial. The steady-state responses of formate species are given by the SSITKA-FTIR. Both, ^12^CO_2_/H_2_ to ^13^CO_2_/H_2_ and ^13^CO_2_/H_2_ to ^12^CO_2_/H_2_, result in identical kinetic responses of ^13^C-labeled formate species (Fig. 2c, blue squares), as expected. Importantly, the response of formate after the switch from pure hydrogen to CO_2_/H_2_ mixture precisely follows the steady-state normalized response of ^13^C-labeled formate. In contrast, the switch from CO_2_/H_2_ back to pure hydrogen results in a completely different, slower response, as compared with the steady-state isotope switch (Fig. 2c, red dots). This observation is one of the central parts of our study, which directly points to the slow hydrogenation of formate over the surface of copper–zinc catalyst, exposed to strongly reducing environment, such as pure hydrogen. Simultaneously, we know from the results of Zn K-edge XANES that high-pressure hydrogen favors the formation of copper–zinc alloy in CZA catalyst (Fig. 2b). Combining these two facts, we conclude that the slow kinetics of the formate decomposition observed under pure hydrogen atmosphere indicates the low activity of copper–zinc alloy in the hydrogenation of formate. In contrast, during the reaction in CO_2_/H_2_ mixture, the oxidative leaching of zinc from the surface copper–zinc alloy yields a surface that is enriched in copper atoms, that are able to split hydrogen yielding fast isotope exchange and formate hydrogenation. Furthermore, the oxidation of the alloy phase plays an essential role by formation of reactive zinc formate species and establishing an intimate contact between zinc phases and the surface of copper, thereby maximizing reactive interphase.

**Fig. 3:**
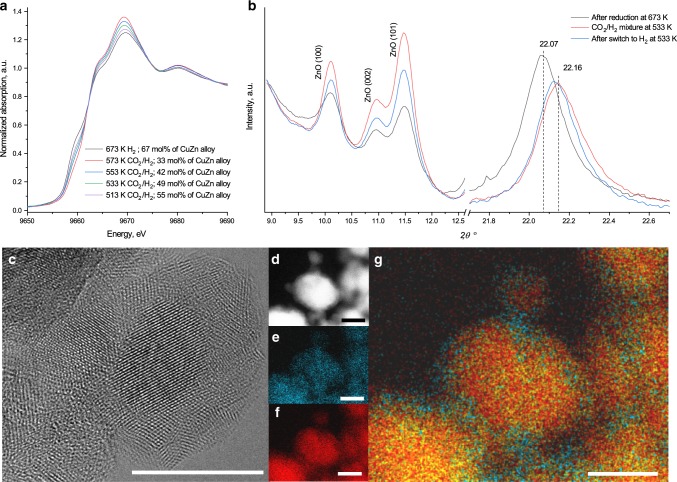
De-alloying of copper–zinc phase under reaction conditions. **a** Zn K-edge XANES spectrum of pretreated at 673 K and 15 bar of hydrogen CZA catalysts as well as spectra of this pretreated material after switch to CO_2_/H_2_ at different reaction temperatures: 573, 553, 533, and 513 K. **b** Operando XRD patterns (λ = 0.4975 A) of CZA catalyst pretreated at 673 K and 15 bar in hydrogen and after switching to CO_2_/H_2_ reaction mixture or back to hydrogen at 533 K. **c** HRTEM micrograph, **d** HAADF-STEM image, EDX mapping of zinc (**e**), copper (**f**), as well as overlay (**g**) after CZA catalyst pretreatment at 673 K and 15 bar in hydrogen and switching to CO_2_/H_2_ reaction mixture at 533 K and reaction for 3 h (scale bar —10 nm).

We further observed that within the time-frame of the experiment identical conditions do not necessary lead to the same structure of the catalyst, if varying pretreatments are used. Figure 3a shows that the initial material reduced at 673 K and containing 67 mol% of zinc in the form of a copper–zinc alloy undergoes partial oxidation into a wurtzite-like zinc oxide phase under reaction conditions. The CZA materials pretreated according to different activation protocols (Table 1) and containing a significantly different amount of reduced zinc (49 and 16 mol%, respectively) show almost identical methanol productivity. A very similar catalytic performance, which correlates well with metallic surface area of copper (Supplementary Table 1, Supplementary Note 4), suggests that the surface composition is (nearly) identical, while the bulk structure is different due to inability of surface-bulk equilibration under reaction conditions. Operando XRD patterns of pretreated CZA catalyst during partial oxidation with CO_2_/H_2_ under reaction conditions (Fig. 3b) clearly demonstrate a shift of copper–zinc (220) diffraction peak from 22.07° to 22.16° (17.4 and 10.7 mol% of zinc in copper–zinc alloy correspondingly) due to a de-alloying process^40^. Concomitantly, (100), (002), and (101) zinc oxide diffraction peaks increase in intensity suggesting reincorporation of zinc into wurtzite (Supplementary Table 2). The copper–zinc surface is, therefore, not stable under the conditions of catalytic carbon dioxide hydrogenation^37,41^. Theoretical calculations (Supplementary Fig. 17, Supplementary Note 5) confirm that the equilibrium between zinc oxide and copper–zinc alloy is very sensitive to the temperature and the gas mixture composition. The calculated Gibbs free energies of formation of zinc oxide surface layers from a copper–zinc alloy under experimental temperatures and carbon dioxide pressure suggest that the higher the temperature during the contact between the in situ prepared copper–zinc alloy and the CO_2_/H_2_ mixture is, the higher the degree of zinc oxidation out of the copper–zinc alloy is expected.

Depending on temperature, we observed different degrees of copper–zinc de-alloying with the formation of the zinc oxide phase (Fig. 3a). Under relatively mild oxidation conditions (513 K) only ≈12 mol% of metallic zinc was oxidized back to zinc oxide, while the main fraction of zinc (55 mol%) remained in the form of copper–zinc alloy. One can assume that oxidation begins at the surface, and therefore one can expect enrichment of the metallic particle surface with copper, concomitant with the occurrence of small zinc oxide particles. TEM, as well as EDX mapping (Fig. 3c and Supplementary Figs. 18–22), confirm the formation of the core–shell ZnO@CuZn structure, which is in good agreement with previously reported data^42^. Detailed analysis of the lattice space distribution suggests the depletion of zinc at the copper–zinc particle surface and the formation of zinc oxide nanoparticles (Supplementary Fig. 20, Supplementary Note 6). Thus, a pretreatment in hydrogen with the formation of copper–zinc alloy, followed by the oxidation with carbon dioxide during catalytic reaction, generates a well-developed interface between zinc oxide and copper, which, in the presence of hydrogen, can activate carbon dioxide and transform it to methanol via a zinc formate intermediate route, as was first suggested by the operando XAS and SSITKA-FTIR techniques. Recent studies^43,44^ highlight the importance of the copper-metal-oxide (titania or zirconia) interface, indicating that both Lewis-acidic sites of oxide and copper nanoparticles are required for formate species formation. Such Lewis acid sites are located on the zinc oxide at the interface with the copper surface.

For a sample re-oxidized in the CO_2_/H_2_ mixture at 573 K (Fig. 3a), zinc oxide nanoparticles with size up to 4 nm were detected (Supplementary Fig. 22), which can be explained by the higher temperature and a higher degree of zinc oxidation. This results in a 25% loss in activity of the catalyst during carbon dioxide hydrogenation to methanol, indicative of the sensitivity of the catalyst performance to minor changes in structure.

## Discussion

Our operando experiments confirm the formation of copper–zinc alloy under highly reducing conditions as it was observed previously^24^. Several research groups have shown recently that it is possible to effectively convert carbon dioxide to methanol by using oxide based systems only: bulk ZnO, ZnO–Cr_2_O_3_, ZnO–ZrO_2_, In_2_O_3_^17,25,36,45–47^. The key factor stressed in these papers was that such materials activate both hydrogen as well as carbon dioxide; however, in the absence of a metallic phase, the activation of molecular hydrogen is significantly limited. Early experiments of Burch et al.^18^. with the physical mixture of Cu/SiO_2_ and ZnO/SiO_2_ suggested that synergetic catalytic activity of the Cu–ZnO system could not be attributed to the formation of the copper–zinc alloy. There, the enhancement in activity of the physical mixture was caused by the spillover of hydrogen, with zinc oxide acting as a reservoir of atomic hydrogen, which then is able to form zinc formate with carbon dioxide. The enthalpy of hydrogen chemisorption over copper–zinc alloy surface is only 9.6 kJ mol^−1^, making the activation of hydrogen unlikely, while pure Cu (111) can activate and split molecular hydrogen more efficiently (Supplementary Table 3). This is in line with the slow formate hydrogenation kinetics observed during the transient switch to pure hydrogen compared with the SSITKA experiment (Fig. 2c), since in the hydrogen atmosphere the zinc oxide phase undergoes reduction to copper–zinc alloy, which inhibits the rate of the formate hydrogenation step. Illustratively, copper–zinc catalysts of similar nature supported over zeolite faujasite (Supplementary Figs. 23 and 24, Supplementary Note 7), which does not reveal the presence of any surface or bulk copper–zinc alloy under CO_2_/H_2_ mixture or hydrogen, nevertheless show high activity and selectivity toward methanol normalized to copper loading (Supplementary Table 4). These data allowed us to suggest a catalytic cycle scheme (Supplementary Fig. 25, Supplementary Note 8). We define active species as all species that are involved in the catalytic cycle. Metallic copper splits hydrogen, which spills over to zinc oxide, leading to the formation of zinc formate after reduction. The formed species undergoes further hydrogenation to methanol, reforming zinc oxide, which enters a new cycle. What is the structure of the reduced zinc oxide phase, i.e., whether it is an oxygen-deficient zinc oxide or a copper–zinc alloy, can neither be conclusively concluded from our experiments nor is obvious from the literature. When the catalyst is exposed to highly reducing conditions, copper–zinc alloy is formed; when the catalyst is exposed to oxygen, the formation of zinc oxide and copper oxide is observed.

In summary, by using operando time-resolved XAS, mass spectrometry, XRD, SSITKA-FTIR and supported by electron microscopy and theory, we were able to unify different, seemingly contradicting, models. These different models are manifestations of the same material, which adopts different structures under varying conditions. Carbon dioxide hydrogenation to methanol occurs on a bifunctional Cu/ZnO catalyst via a zinc formate intermediate route. The catalyst transforms completely into the copper–zinc alloy, only under highly reducing atmospheres. However, it is unstable in the presence of carbon dioxide, and its surface undergoes oxidation with the formation of zinc oxide and surface zinc formate, which is the reactive intermediate. The preformation of a copper–zinc alloy and subsequent zinc oxidation yields a highly abundant copper–zinc oxide interface, where the reaction takes place. Intimate contact between nano-sized zinc oxide and the copper phases facilitates the hydrogenation of the formate species by hydrogen, which is dissociated on the metallic copper phase. The results of this mechanistic investigation are a prototype of the unique interplay between different components in a heterogeneous catalyst and provide a guideline for the catalyst improvement and/or the discovery of the new class of catalysts to make this important process commercially viable.

## Methods

### Commercial CZA catalyst

Copper-based methanol synthesis catalyst (product number: 45776; lot number I06Z036) containing 10.1 wt% Al_2_O_3_, 63.5 wt% CuO, 24.7 wt% ZnO and 1.3 wt% MgO was purchased from Alfa Aesar.

### CuZn-FAU catalyst

CuZn-FAU catalyst was prepared accordingly to the following synthesis protocol from commercially available faujasite (SiO_2_/Al_2_O_3_ = 12, CBV712, Zeolyst). In a first step, ammonium form of CBV712 was ion exchange with copper. One gram of zeolite was dispersed in 100 ml of 0.1 M solution of copper nitrate (99%, Sigma-Aldrich) and stirred overnight at 323 K. After that sample was filtered, thoroughly washed with 500 ml of deionized water, and dried at 393 K for 1 h. On the next step, prepared material was dispersed in 500 ml of 0.01 M solution of sodium carbonate (99%, Sigma-Aldrich) and stirred at 323 K for 5 h. The resulting sample was filtered, thoroughly washed with deionized water until pH = 7, dried at 393 K overnight, and calcined at 623 K for 4 h in a flow of dry synthetic air. Prepared material containing copper oxide nanoparticles encapsulated inside sodium form of faujasite was further dispersed in 100 ml of 0.1 M solution of zinc nitrate (99%, Sigma-Aldrich) and stirred overnight at 323 K. After that, sample was filtered, thoroughly washed with 500 ml of deionized water, and dried at 393 K for 1 h. After that, prepared material was dispersed in 500 ml of 0.01 M solution of sodium carbonate (99%, Sigma-Aldrich) and stirred at 323 K for 5 h. The resulting sample was filtered, thoroughly washed with deionized water until pH = 7, dried at 393 K overnight, and calcined at 623 K for 4 h in a flow of dry synthetic air. Synthesized material containing 2.6 wt% Cu and 2.4 wt% Zn (determined by AAS) was marked as CuZn-FAU and was used as synthesized during catalytic and XAS experiments.

### One weight percent Cu/ZnO catalyst

In total, 45.6 mg of copper nitrate trihydrate (99% Sigma-Aldrich) and 4.15 g of zinc nitrate hexahydrate (99%, Sigma-Aldrich) were dissolved in 30 ml of deionized water. After that, abovementioned solution was added to 100 ml of 0.2 M solution of sodium carbonate (99%, Sigma-Aldrich) pre-heated to 323 K under vigorous stirring. During addition slightly blue precipitate was formed. Suspension was kept stirring for 1 h at 323 K and after that the precipitate was filtered, thoroughly washed with deionized water, dried overnight at 393 K, and finally calcined at 623 K for 4 h in a flow of dry synthetic air (heating ramp 2 K min^−1^). Synthesized material containing 1.1 wt% Cu (determined by AAS) was marked as 1 wt% Cu/ZnO and was used as synthesized during catalytic and XAS experiments.

### Operando XAS and XRD spectroscopies

Time-resolved operando XAS measurements were performed at the SuperXAS beamline, Swiss Light Source of the Paul Scherrer Institute in Villigen, Switzerland. Combined operando XRD and XAS study was performed at the Swiss-Norwegian BM31 beamline, the European Synchrotron Radiation Facility (ESRF, Grenoble, France)^48^. About 1 mg of commercial CZA catalyst (fraction 50–100 μm) diluted with 5 mg of boron nitride was positioned inside the 1 mm, (wall thickness 0.01 mm) quartz capillary reactor and fixed between two quartz wool plugs. A modified version of the plug-flow reactor system developed by Chupas et al. for total X-ray scattering measurements was used during these experiments^49^. All measurements made with this reactor system utilized 0.3 mm a K-type thermocouple inserted into the sample bed. Gas flows (2–10 ml min^−1^) were controlled by mass flow controllers (Bronkhorst). The total pressure was controlled by Bronkhorst EL-Press digital back-pressure regulator. All gases (H_2_, CO_2_, Ar, and He) of grade 6.0 were used. Transient switches were performed by using remotely controlled 6 port 2-position valve (VICI, Valco Instruments).

The X-ray beam, sample, and X-ray detectors were in conventional transmission geometry. A quick scanning channel-cut Si(111) monochromator with oscillation frequency of 10 Hz was used for time-resolved XAS measurements at SuperXAS beamline. A high-resolution powder diffraction-dedicated channel-cut monochromator and a second double crystal monochromator for XAFS were used during combined XAS and XRD study at SNBL BM31 beamline. A complementary metal-oxide semiconductor detector with an active area of 290.8 × 229.8 mm was used during synchrotron X-ray powder diffraction experiment^50^. Spectra of copper and zinc foil were collected simultaneously for internal energy calibration. Transient responses of reaction mixture and products during switches experiments were monitored by means of Omnistar GSD 300 O2 (Pfeiffer Vacuum) mass spectrometer.

### Analysis of XANES and EXAFS data

Initial analysis and energy calibration were performed using ProXAS v.2.34 software^51^. Cu K- and Zn K-edge XANES and EXAFS data were background subtracted and normalized using either, PAXAS^52^, Athena^53^, or Prestopronto^54^. PCA was made using the ITFA software due to Rossberg et al.^55^. Fitting of the EXAFS was made using EXCURV (v. 9.3)^56^.

### Operando SSITKA-FTIR experiments

SSITKA experiments were performed using standard flow reactor configuration with sandwich-type transmittance IR cell^57^. For all experiments, the sample was diluted with pure silica (1:3 by weight). After achieving steady-state methanol conversion (ca. 1 h on stream), isotopic switch was performed using a two-position valve (VICI), from unlabeled ^12^CO_2_/H_2_ (Messer; 99.5%) to ^13^CO_2_/H_2_ labeled mixture (Cambridge Isotopes Laboratories, Inc.; 99% ^13^C). The ^12^C-labeled reagents gas feed was mixed with an inert argon tracer (4 vol% of the total flow rate) that was used to correct for the gas-phase holdup in the reactor. The SSITKA experiments were carried out at constant total gas flow rates of 25 cm^3^ min^−1^. The ^12^C/^13^C switch was achieved without perturbing the steady-state of the reaction by maintaining the reaction temperature at 533 K, the total system pressure at 15 bar and carbon dioxide conversion at ≈2%. On-line mass spectrometry analysis was performed by quadrupole mass analyzer (Balzers). The surface species on the catalyst were followed using IR spectroscopy (Thermo Nicolet iS50 equipped with MCT detector). Spectra resolution was set to 4 cm^−1^, spectra were acquired each 6 s with 4 scans per each spectrum.

In the MS, the ion signals for *m*/*z* = 40 (Ar tracer), 44, 45, 28, 29, 31, and 33 were continuously monitored to determine the isotope content of the original gas sample. Due to the overlapping of MS signals of CO and CO_2_, the ^12^CO and ^13^CO responses were separately analyzed: the gas feed passing through the trap filled with the solid sodium hydroxide, which absorbed all CO_2_. Transient responses were normalized by the difference between the initial and final ion signals. The argon decay curve was used to determine the gas-phase holdup of the reactor system, since we assumed that the inert gas did not adsorb on the surface of the catalyst.

### TEM microscopy

Particle size and morphology were studied by means of scanning TEM with a high-angle annular dark field detector (HAADF-STEM) and high-resolution transmission electron microscopy (HRTEM). Samples were transferred to TEM under ambient air conditions. HAADF-STEM imaging and elemental mapping based on energy-dispersive X-ray spectroscopy was performed on a probe-corrected HD2700CS (Hitachi) at 200 kV. For HRTEM, a double aberration-corrected JEM-ARM300CF (JEOL) working at 300 kV was employed. The species observed in the TEM micrograph are obtained after exposure to air and represent the case of not catalytic conditions indicated in Supplementary Fig. 22.

### Catalytic experiments

Catalytic carbon dioxide hydrogenation over commercial CZA catalyst was tested in a fixed-bed stainless-steel reactor using 25 mg of catalyst (fraction 50–100 μm), diluted with 75 mg of SiC. Powdered catalyst was positioned inside the 4 mm I.D. stainless-steel tube (wall thickness 1 mm) and fixed between two quartz wool beads. The reactor tube was mounted inside of a single-zone furnace. Temperature was controlled using a K-type thermocouple positioned inside the catalyst-bed. Catalysts were firstly in situ pretreated in a flow of Ar (50 mL min^−1^) at 533 K and ambient pressure for 2 h (heating rate 5 °C min^−1^) and after that reduced in H_2_ flow (50 mL min−^1^, 15 bar) at 533 or 673 K (see figure captions and information in the tables). Total pressure was controlled by back-pressure regulator (Bronkhorst, EL-press series). Catalytic CO_2_ hydrogenation was performed at 15 bar total pressure and 533 K. Feed gas mixture during catalytic experiment contained 24 vol% of carbon dioxide, 72 vol % of hydrogen and 4 vol% of argon (tracer and internal standard), while the gas flow rate was equal to 50 mL min^−1^ (controlled by Bronkhorst mass flow-controller). Conversion of carbon dioxide was in the range 2–5%. Analysis of outlet gases was performed by gas chromatography using a 3000 Micro GC gas analyzer (Inficon) equipped with 10 m Molsieve and 8 m PlotU columns and TCD detectors. Transient switches were performed by using remotely controlled 6 port 2-position valve (VICI, Valco Instruments). Transient responses of reaction mixture and products during switches experiments were monitored by means of an Omnistar GSD 300 O2 (Pfeiffer Vacuum) mass spectrometer.

### O_2_-chemisorption experiments

In order to estimate metallic copper surface area of CZA samples after catalytic examination where different pretreatment protocols were applied (see Table 1 for details) oxygen chemisorption experiments were done. The measurement was performed in a Micromeritics 3Flex instrument using 3Flex V 4.04 software. Prior to chemisorption measurement, the investigated material was in situ evacuated to 5·10^−3^ mmHg at 393 K for 30 min and then reduced in flow of hydrogen (purity 5.0) at 423 K and ambient pressure for 1 h. After that investigated material was evacuated to 1·10^−4^ mmHg at 423 K for 30 min in order to desorb any hydrogen. Then the temperature was lowered to 308 K and the total adsorption isotherm (chemisorption + physisortpion) of oxygen was collected by the pulse chemisorption of oxygen (dose amount—2 cm^3^ g^−1^). After measuring the total oxygen adsorption isotherm, the sample was evacuated to 1·10^−4^ mmHg and the second adsorption isotherm was collected in order to determine the amount of physically adsorbed oxygen. The amount of surface copper atoms and metallic surface area of copper were calculated using difference method (3Flex V 4.04 software) assuming a stoichiometry factor of 4 (one irreversibly adsorbed molecule of oxygen per 4 surface atoms of copper).

### Density functional theory calculations methodology

All ground-state total energy calculations in this work have been performed with the all-electron full-potential DFT code FHI-aims^58,59^ within the periodic boundary conditions model. Electronic exchange and correlation was treated on the GGA functional level with the PBE functional^60^. All geometry optimization were done with the tier2 atom-centered basis set using tight settings for numerical integrations. Tkatchenko–Scheffler dispersion correction^61^ has been used to account for the van der Waals energies arising from the attraction between induced dipoles formed due to charge fluctuations in the interacting species. All geometries reported herein correspond to the locally optimized intermediate configurations.

All energy values are computed as free Gibbs energies corresponding to the experimental temperatures and pressures. In the spirit of ab initio thermodynamics^62^, we calculated the translational, rotational, and vibrational contributions to the Gibbs free energy according to the following formula:

1 $$G\left( {T,p} \right) = \, G\left( {{\mathrm{electr}}.} \right) + \mathop {\sum }\limits_i \left\{ {N_A\frac{{h\nu _i}}{{4\pi }} + RT\ln \left[ {1 - \exp \left( {\frac{{ - h\nu _i}}{{k_BT}}} \right)} \right]} \right\} \\ - \, RT\ln \left( {\frac{{\left( {2\pi k_BTm} \right)^{\frac{3}{2}}}}{{h^3}} \cdot \frac{{k_BT}}{p}} \right) - RT\ln \left[ {\frac{{8\pi ^2}}{\sigma }\left( {\frac{{2\pi k_BT}}{{h^2}}} \right)^{\frac{3}{2}} \cdot \,\left( {I_AI_BI_C} \right)^{\frac{1}{2}}} \right],$$

with the first term of the vibrational free energy accounting for zero-point vibrations. The translational, rotational, and vibrational contributions are easily calculated from the structural data and the calculated vibrational spectrum. The *G*(electr.) term is then equal to the calculated total energy of the system.

### Gibbs free energies of formation

In order to calculate the thermal effects of alloying, Zn oxidation and reduction (Supplementary Fig. 17), the following processes were considered (only schemes without exact stoichiometry are presented below):

2 $${\mathrm{CuO}}\left( {{\mathrm{bulk}}} \right) + {\mathrm{ZnO}}\left( {{\mathrm{bulk}}} \right) + {\mathrm{H}}_2 \to {\mathrm{CuZn}}\left( {{\upalpha }} - {\mathrm{brass}} \right) + {\mathrm{H}}_2{\mathrm{O}}.$$

3 $${\mathrm{CuZn}}\left( {{\upalpha }} - {\mathrm{brass}} \right) + {\mathrm{CO}}_2 \to {\mathrm{ZnO}}@{\mathrm{Cu}}/{\mathrm{Zn}} + {\mathrm{CO}}.$$

4 $${\mathrm{ZnO}}@{\mathrm{Cu}}/{\mathrm{Zn}} + {\mathrm{H}}_2 \to {\mathrm{CuZn}}@{\mathrm{ZnO}}@{\mathrm{CuZn}} + {\mathrm{H}}_2{\mathrm{O}}.$$

For each reaction, experimental temperatures and pressures were considered.

## Supplementary information

Supplementary Information

Peer Review File

## Data Availability

All data needed to evaluate the conclusions are presented in the paper and in Supplementary information file. All RAW data generated during this study are stored on the internal servers of Paul Scherrer Institute and are available from the corresponding authors upon reasonable request.
