# Supplementary material for: The unique interplay between copper and zinc during catalytic carbon dioxide hydrogenation to methanol
Source: Nat Commun. 2020 May 15;11:2409. doi: 10.1038/s41467-020-16342-1 (PMC7229192; doi:10.1038/s41467-020-16342-1)
Supplement: Supplementary file 1 — Supplementary Information [file 41467_2020_16342_MOESM1_ESM.pdf]

Supplementary Information for

The unique interplay between copper and zinc during catalytic carbon dioxide hydrogenation to methanol

Zabilskiy et al.

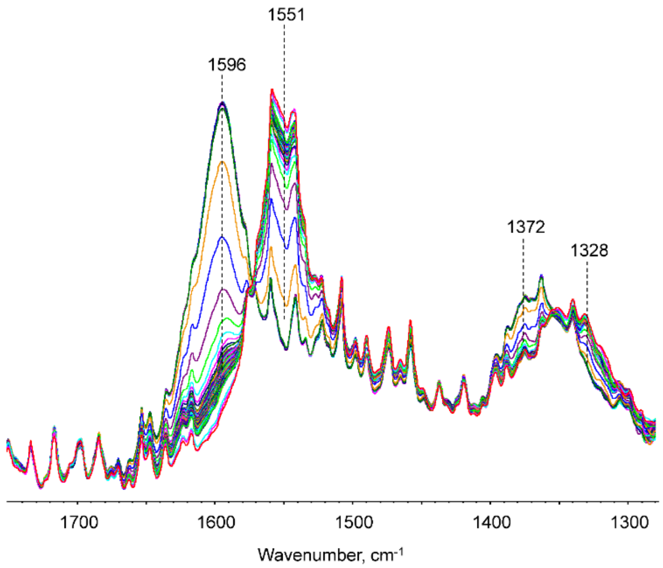

Supplementary Fig. 1. Time-resolved in situ FTIR spectra of surface species formed during switch from $^{12}CO_2/H_2$ to $^{13}CO_2/H_2$ mixture at 533 K and 15 bar during the reaction over CZA catalyst diluted with silica.

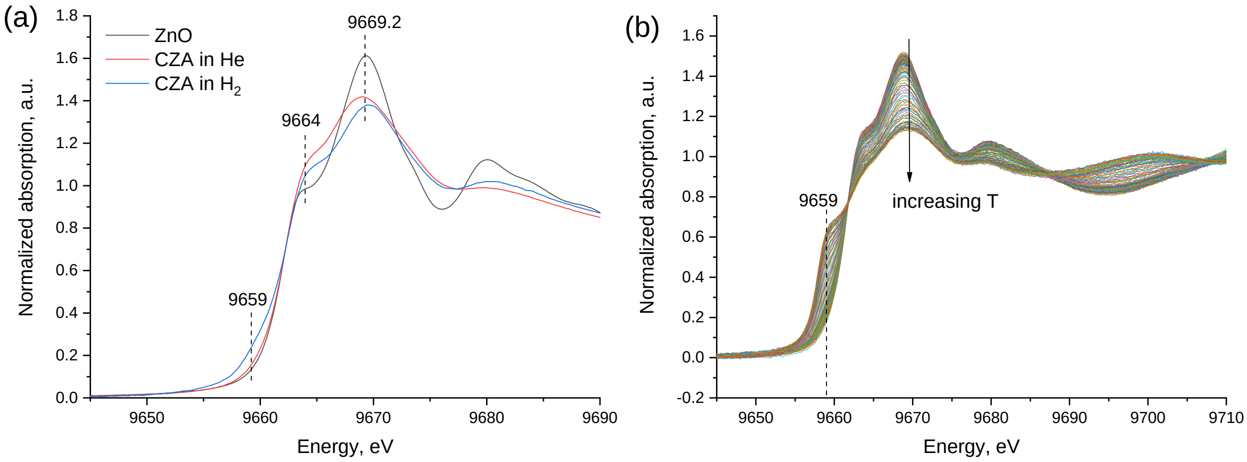

Supplementary Fig. 2. (a) Comparison of experimental spectra of ZnO wurtzite standard (black), CZA catalyst after pre-treatment in He at 533 K (red) and CZA catalyst after pre-treatment in H_2 at 533 K and 15 bar (blue). (b) Evolution of Zn *K*-edge XANES during H_2-TPR-XAS experiment.

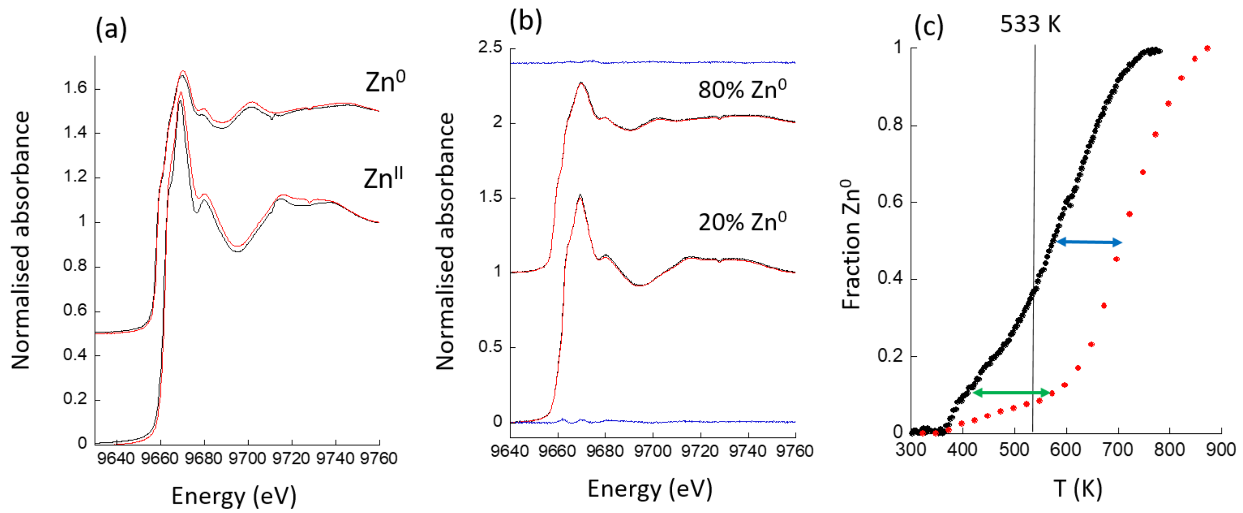

Supplementary Fig. 3. (a) Spectroscopic components (Zn^{II} and Zn^0, as indicated) extracted by PCA analysis from Zn K-edge XANES made during temperature programmed reduction under H_2 (red) and compared to Zn *K*-edge XANES derived from two standard materials (ZnO-wurtzite- and a $Cu_{0.67}Zn_{0.33}$ foil). (b) Comparison of experimental and PCA calculated Zn K-edge XANES data for two degrees (20% and 80%) of Zn reduction obtained at different points in the TPR made under 15 bar H_2. The black lines are the experimental data, the red, the PCA reproduced spectra. The blue lines are the residual differences between the two. (c) Temperature dependence of the evolution of the Zn^0 phase during TPR under 1 bar H_2 (red symbols) and 15 bar H_2 (black symbols). The methanol synthesis reaction temperature (533 K) is indicated by the vertical black line. The horizontal blue line (indicating a difference in temperature of ca. 150 K) highlights the differences in degree of Zn reduction achieved at the methanol synthesis reaction temperature between the two cases. The horizontal green line shows the difference in temperature (ca. 125 K) required to achieve 10% reduction of the Zn^{II} during the first of the two stages of reduction indicated to be present.

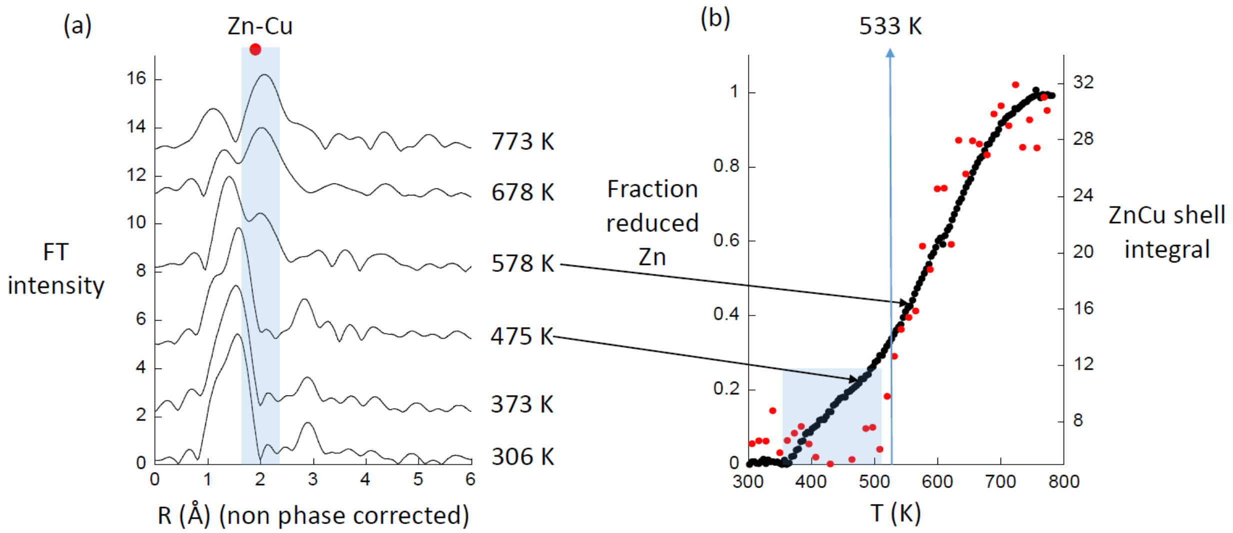

Supplementary Fig. 4 (a) Fourier transforms of the k^3-weighted Zn *K*-edge EXAFS obtained during TPR of the catalyst under 15 bar H_2. The blue shaded region indicates the range of r (Å) space used to obtained the ZnCu shell integral shown in Supplementary Fig. 4 (b); (b) Left hand ordinate (black symbols): Fraction of reduced Zn derived from PCA analysis; Right hand ordinate (red symbols): the variation in the integral in the Fourier transform corresponding to CuZn scattering and as delineated by the shaded blue rectangle in Supplementary Fig. 4 (a). The shaded blue are in Supplementary Fig. 4 (b) shows the range of temperatures/Zn reduction wherein Zn reduction is evident in the Zn *K*-edge XANES but where no significant evidence for a CuZn scattering interaction is present in the Zn *K*-edge EXAFS. The methanol synthesis reaction temperature (533 K) is also indicated.

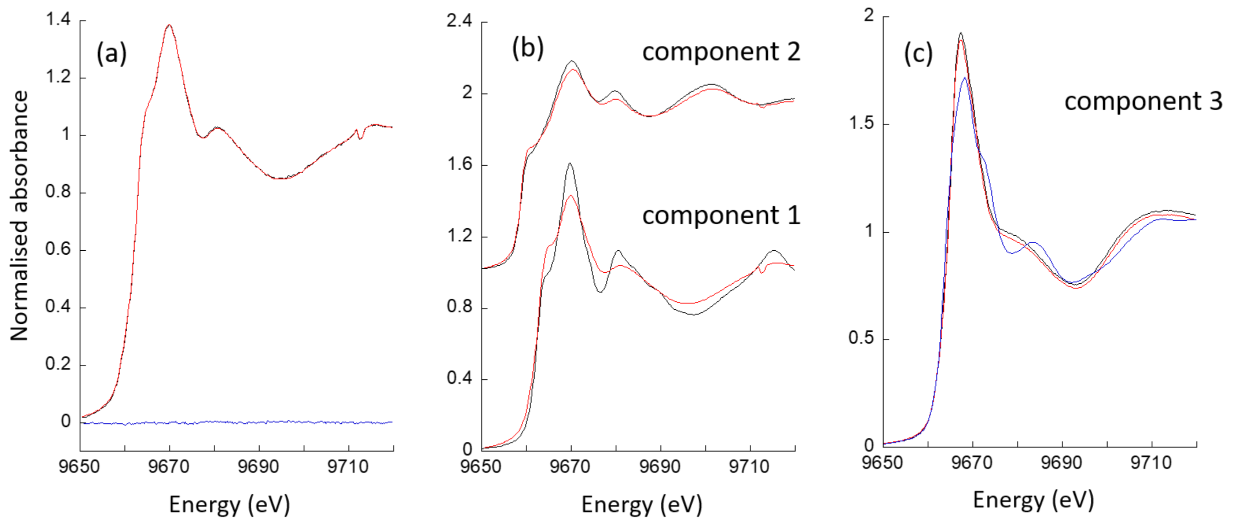

Supplementary Fig. 5. (a) An example of the quality of spectral reproduction obtained from PCA analysis of Zn K-edge XANES obtained during a switch from 15 bar H_2 to 15 bar CO_2/H_2 at 533 K: black = experiment; red = PCA obtained spectrum; blue = residuals. (b) Comparison of components 1 and 2 derived from PCA analysis to bulk standards: component 1 = oxidized Zinc (ZnO, wurtzite); component 2 = reduced zinc ($Cu_{0.67}Zn_{0.33}$ alpha-brass foil). In each case the black curve is that derived from the bulk standard, the red from the PCA analysis. (c) Comparison component 3 (red) derived from the PCA analysis to the Zn Kedge XANES derived from solid zinc formate (black) and zinc-hydroxy-carbonate (blue).

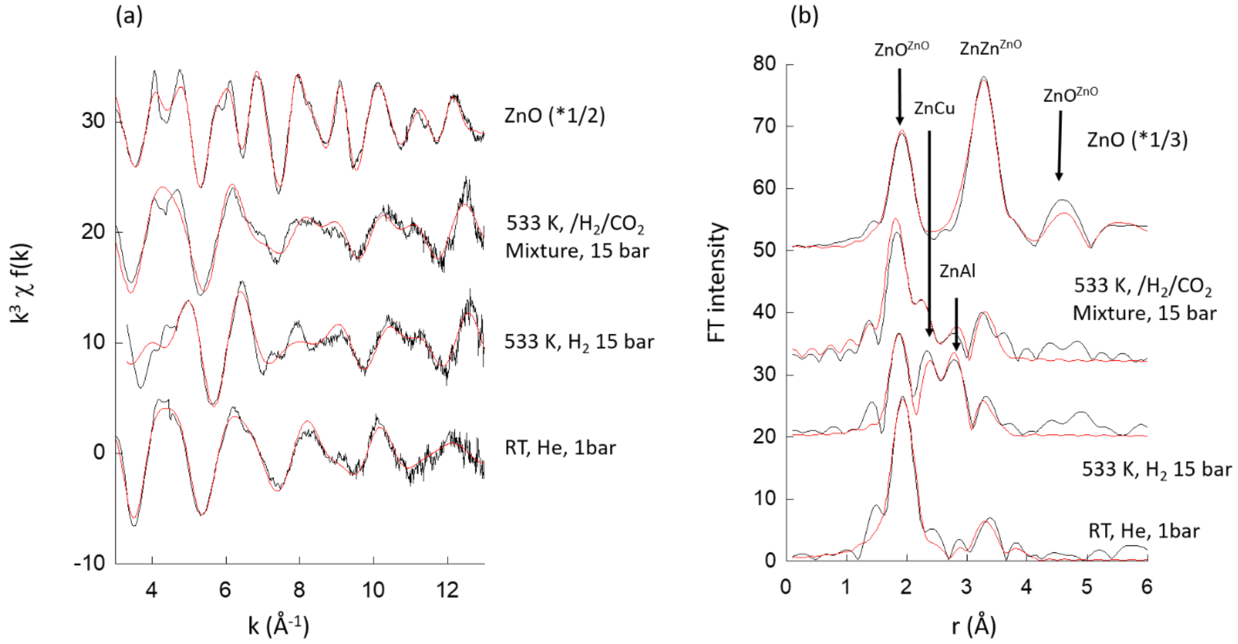

Supplementary Fig. 6 (a) k^3-weighted Zn *K*-edge EXAFS derived from the catalyst under He at ambient temperature; reduced at 673 K and 15 bar H_2 and measured under 15 bar H_2 at 533 K; at 533 K under a catalytic mixture of CO_2 and H_2 at 15 bar. The k^3-weighted EXAFS derived from a bulk ZnO (wurtzite) standard measured at RT is also given. (b) Shows the corresponding phase-corrected Fourier transforms of the k^3-weighted EXAFS in each case.

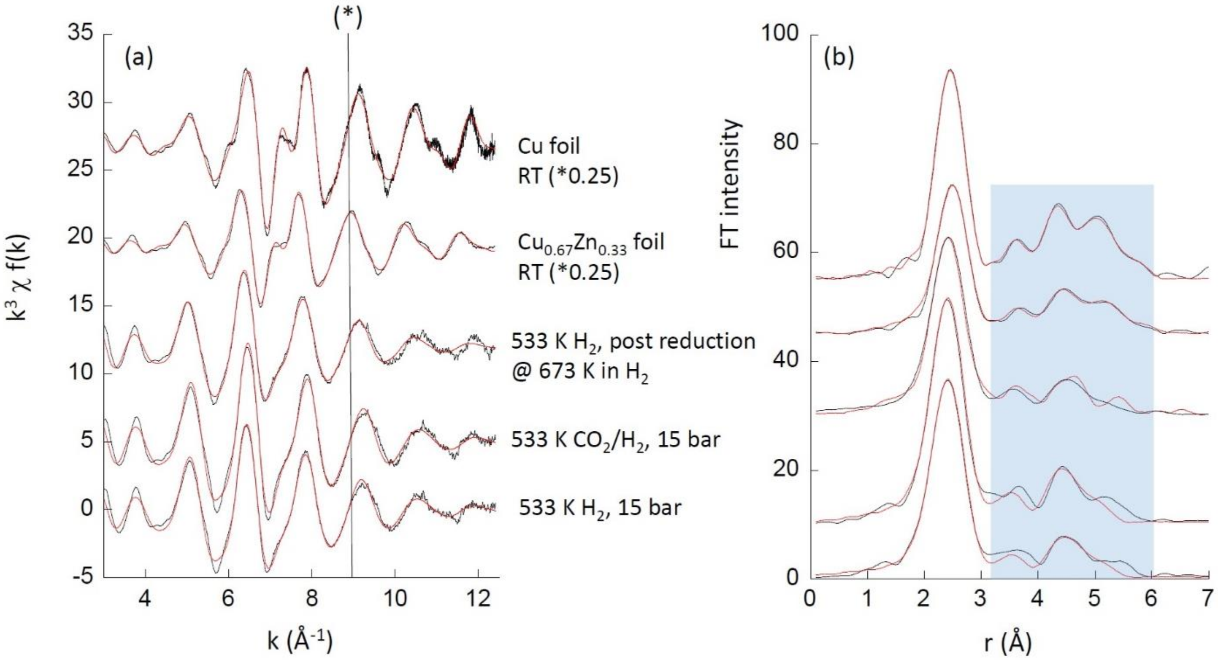

Supplementary Fig. 7 (a) k^3-weighted copper K edge EXAFS derived from the catalysts in three steady state conditions: under 15 bar H_2 at 533 K; under 15 bar CO_2/H_2 catalysis mixture at 533 K; and under 15 bar H_2 again at 533 K post reduction in H_2 to 673 K. The top two spectra correspond to the EXAFS derived from copper and copper-zinc alpha-brass ($Cu_{0.67}Zn_{0.33}$) reference foils measured at ambient temperature The vertical back line and (*) serve to guide the eye toward the difference sin phase observed in the EXAFS in each of the cases shown. (b) The corresponding Fourier transforms of the k^3-weighted Cu K-edge EXAFS data. The shaded blue area highlights the higher shell structure and changes that occur within it.

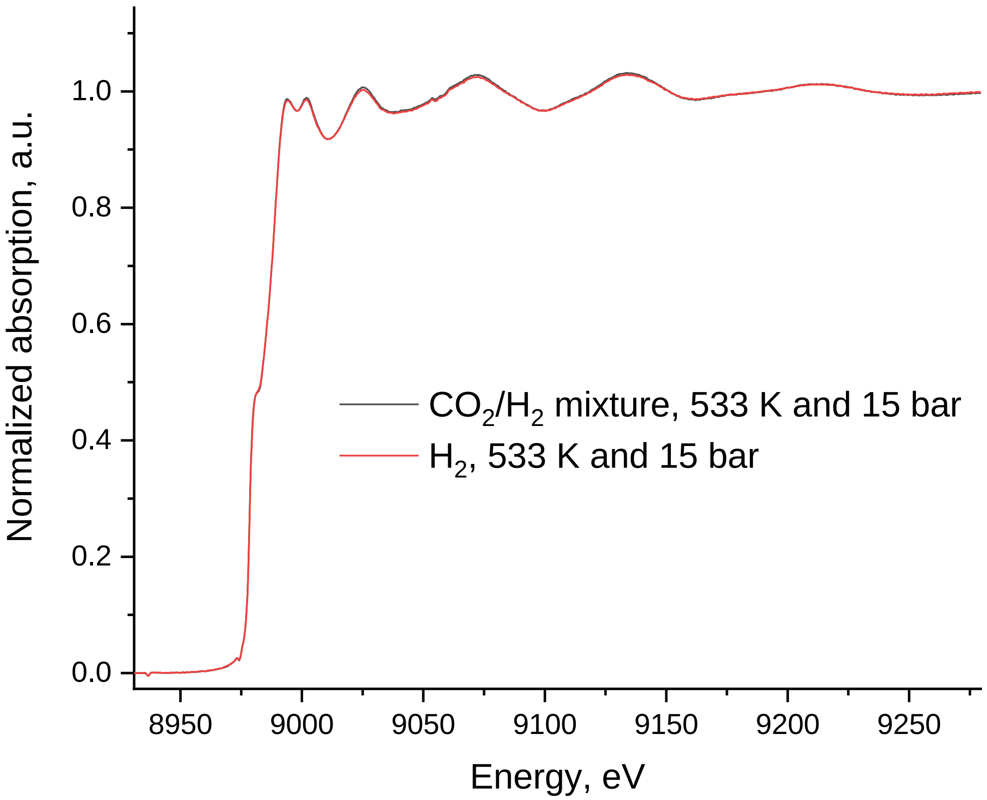

Supplementary Fig. 8 Cu *K*-edge XANES spectrum of CZA catalysts under operando conditions, as well as spectrum of this material after switch to H_2 at 533 K and 15bar.

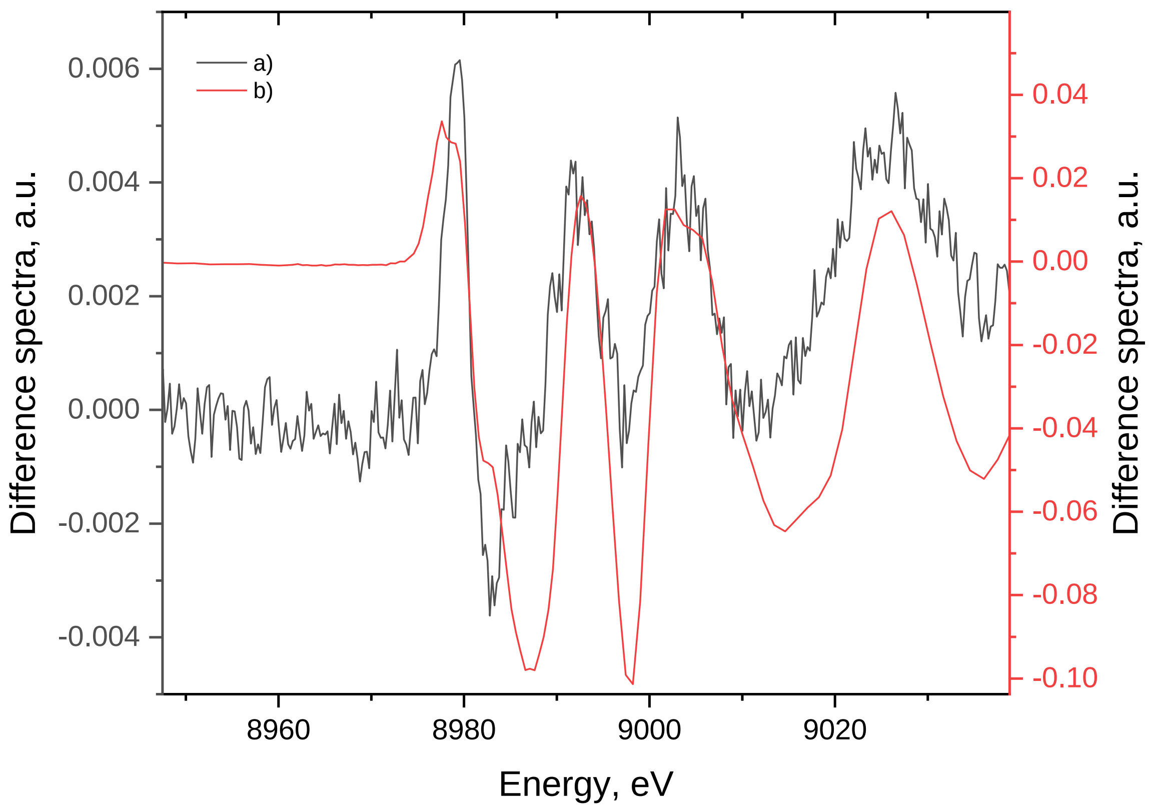

Supplementary Fig. 9. Difference spectrum between CZA catalyst under CO_2/H_2 atmosphere and the same catalyst in hydrogen (black), as well as difference spectrum between copper foil and copper zinc alloy foil standards (red).

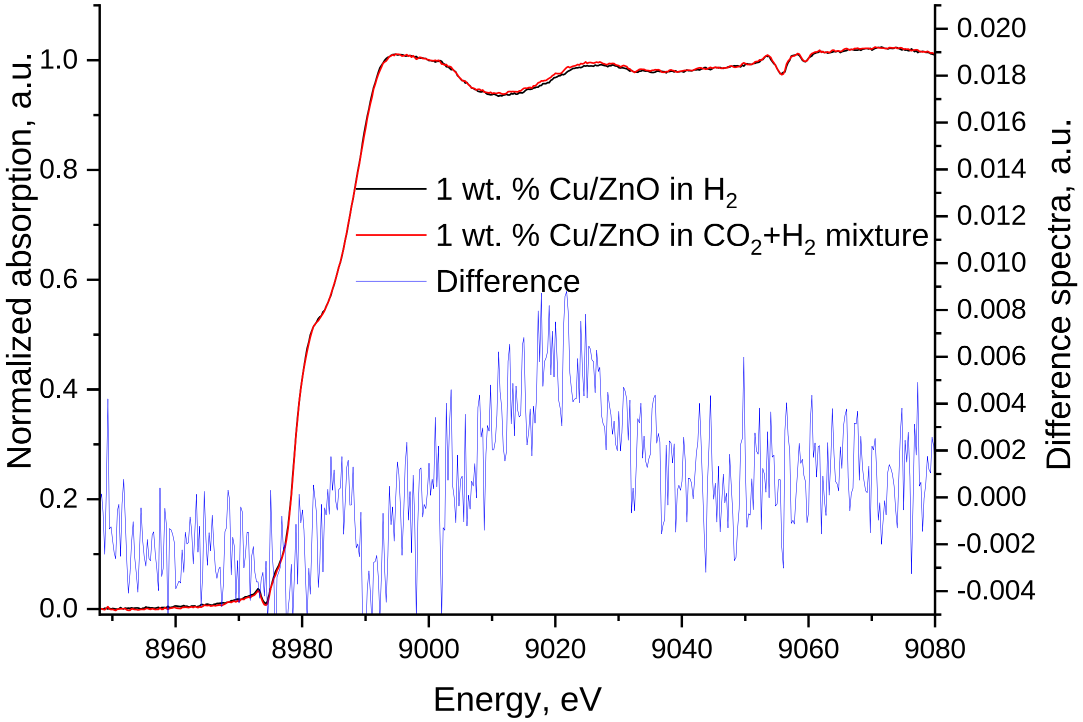

Supplementary Fig. 10. Cu K edge XANES of 1 wt. % copper supported on zinc oxide under hydrogen atmosphere (black) and after transient switch to CO_2/H_2 mixture (red) at 15 bar and 533 K; as well as difference spectrum (blue) showing absence of any oxidation processes due to formate formation over copper surface.

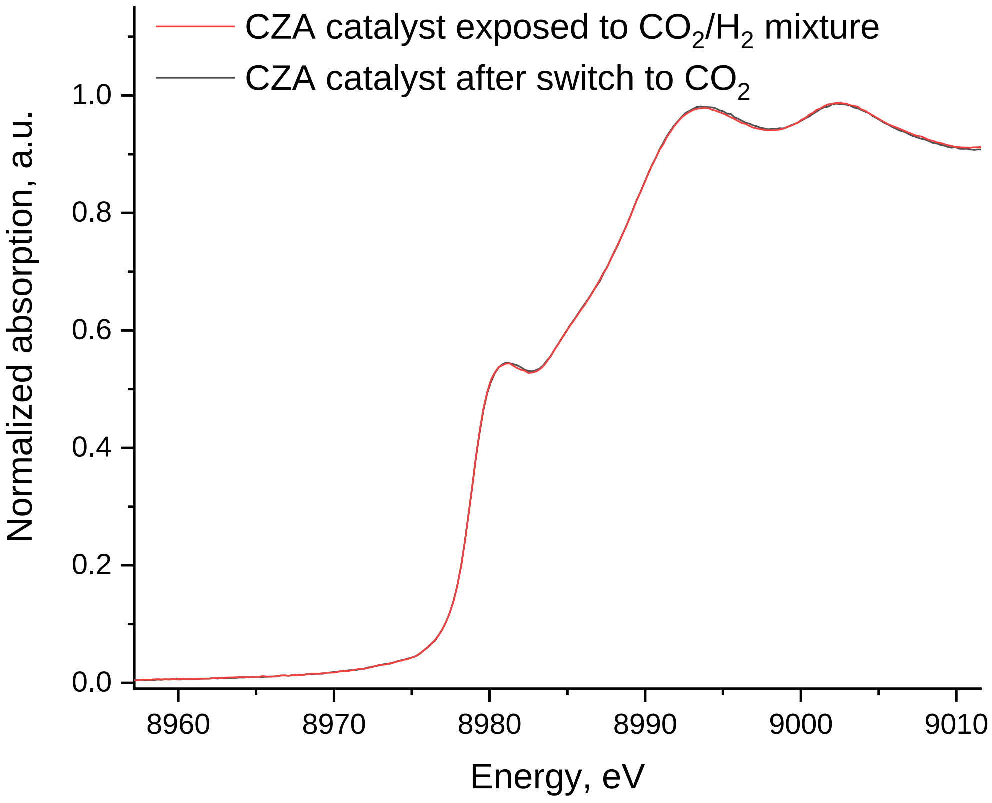

Supplementary Fig. 11. Cu K-edge XANES of CZA catalyst during catalytic methanol synthesis conditions (CO_2/H_2 mixture 3:1; 533 K and 15 bar) and after switch to pure carbon dioxide at 533 K and 15 bar.

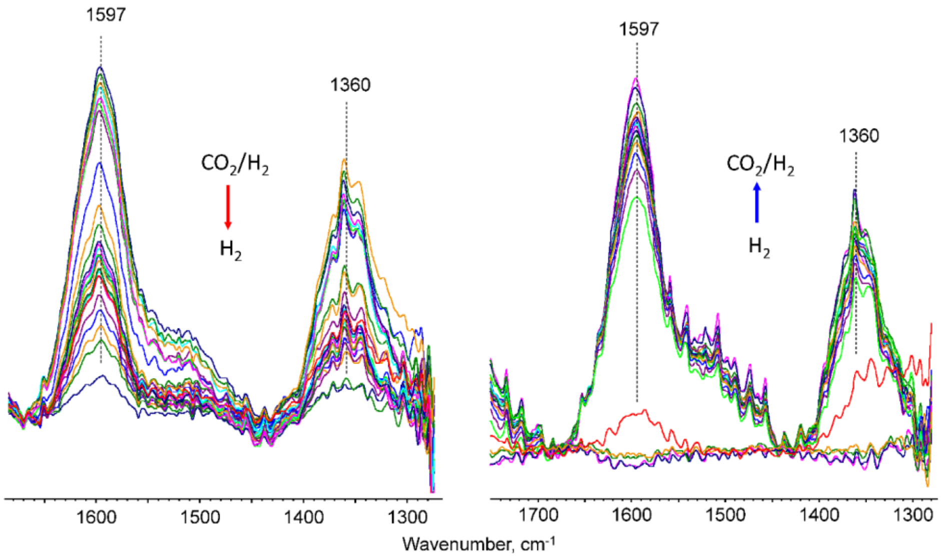

Supplementary Fig. 12. Time-resolved in situ FTIR spectra evolution of surface species during switch from hydrogen to $^{12}CO_2/H_2$ mixture and from $^{12}CO_2H_2$ mixture to hydrogen at 533 K and 15 bar during over CZA catalyst diluted with silica.

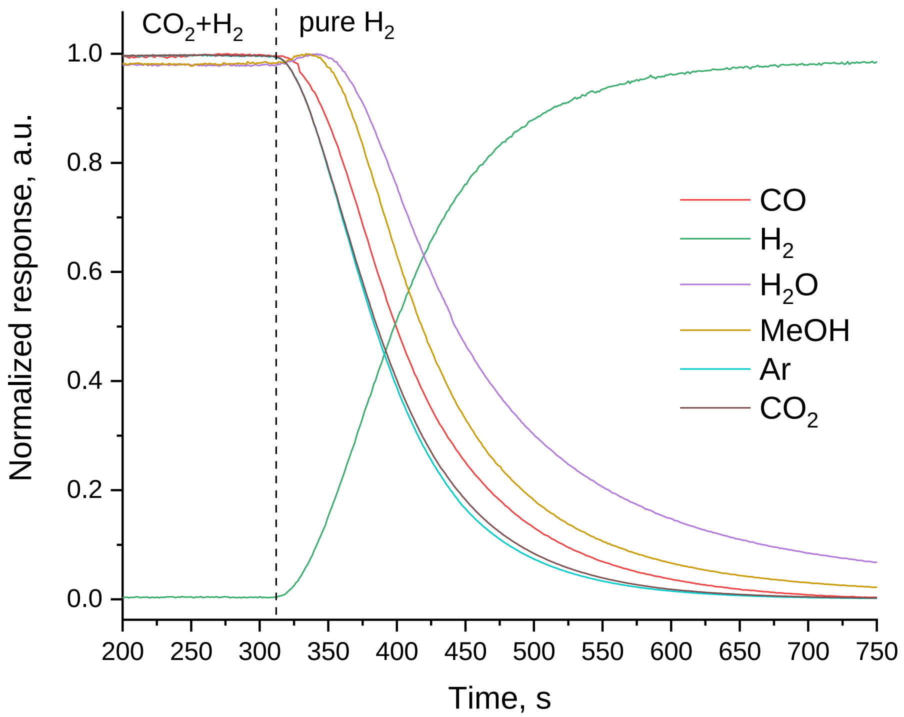

Supplementary Fig. 13. Normalized MS-response curves following the switch from CO_2/H_2 mixture (H_2/CO_2 molar ratio kept 3; 4 vol. % of Ar was used as a tracer) to pure H_2 with a total flow of 50 cm^3 min^{-1}, at 533 K and 15 bar during the reaction over CZA catalyst diluted with SiC.

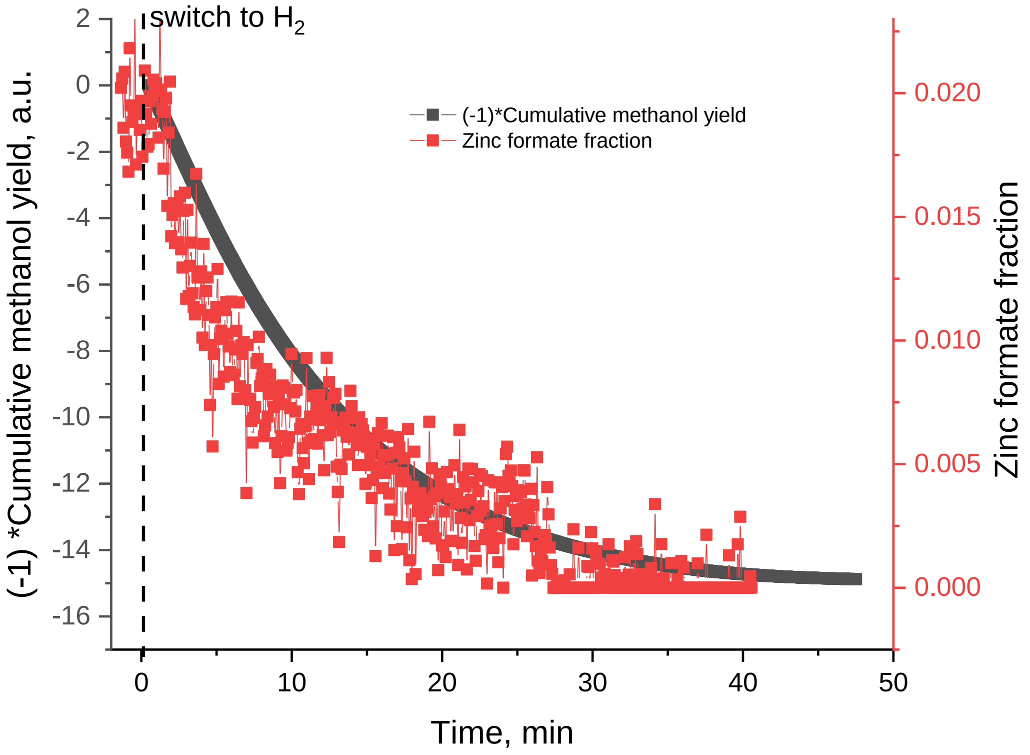

Supplementary Fig. 14. (-1) multiplied by cumulative methanol yield following the switch from CO_2/H_2 to pure hydrogen with a total flow of 10 cm^3 min^{-1}, at 533 K and 15 bar during the coupled operando XAS and MS study (black curve) as well as fraction of zinc formate following the switch determined by XANES-PCA analysis of operando Zn K-edge XANES.

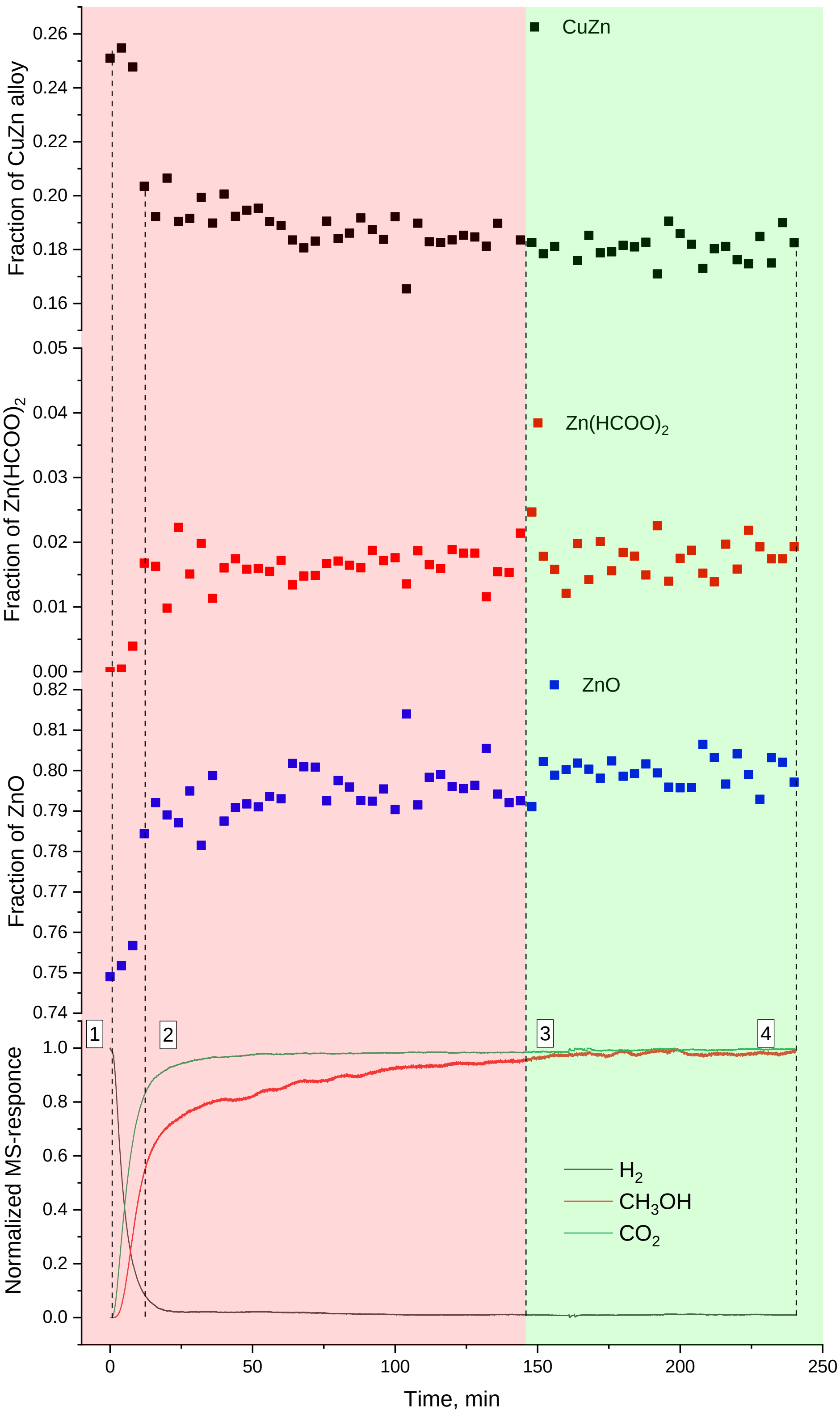

Supplementary Fig. 15. The relative fractions of copper-zinc alloy, zinc formate and zinc oxide wurtzite determined from PCA analysis of XANES spectra as function of time after switch from hydrogen to CO_2/H_2 mixture at 533 K and 15 bar over CZA catalyst as well as normalized MS-response curves of hydrogen, methanol and carbon dioxide following the abovementioned switch. With dot lines (1-4) indicating different points of time where Zn K-edge spectra were additionally compared (see Supplementary Fig. 16).

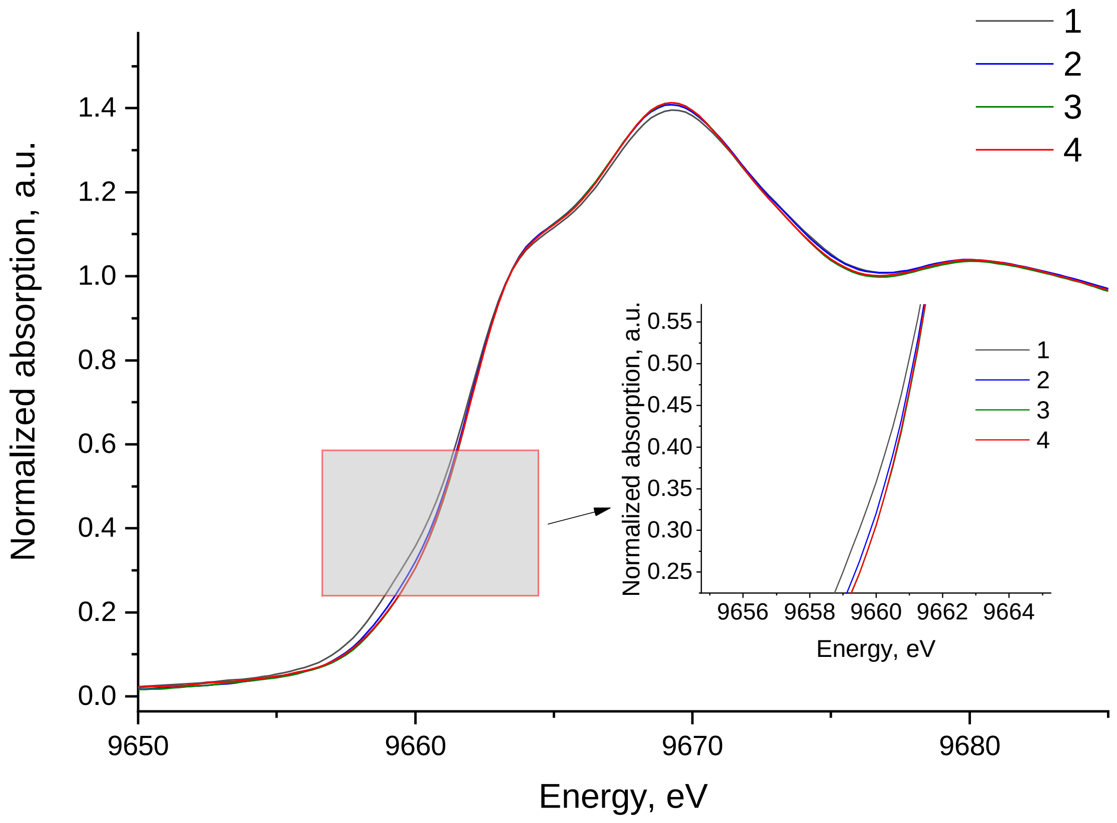

Supplementary Fig. 16. Zn K-edge XANES spectra of CZA catalyst during *operando* switch from pure hydrogen to CO_2/H_2 gas reaction mixture at 15 bar and 533K acquired at different points of time indicated on Supplementary Fig. 15.

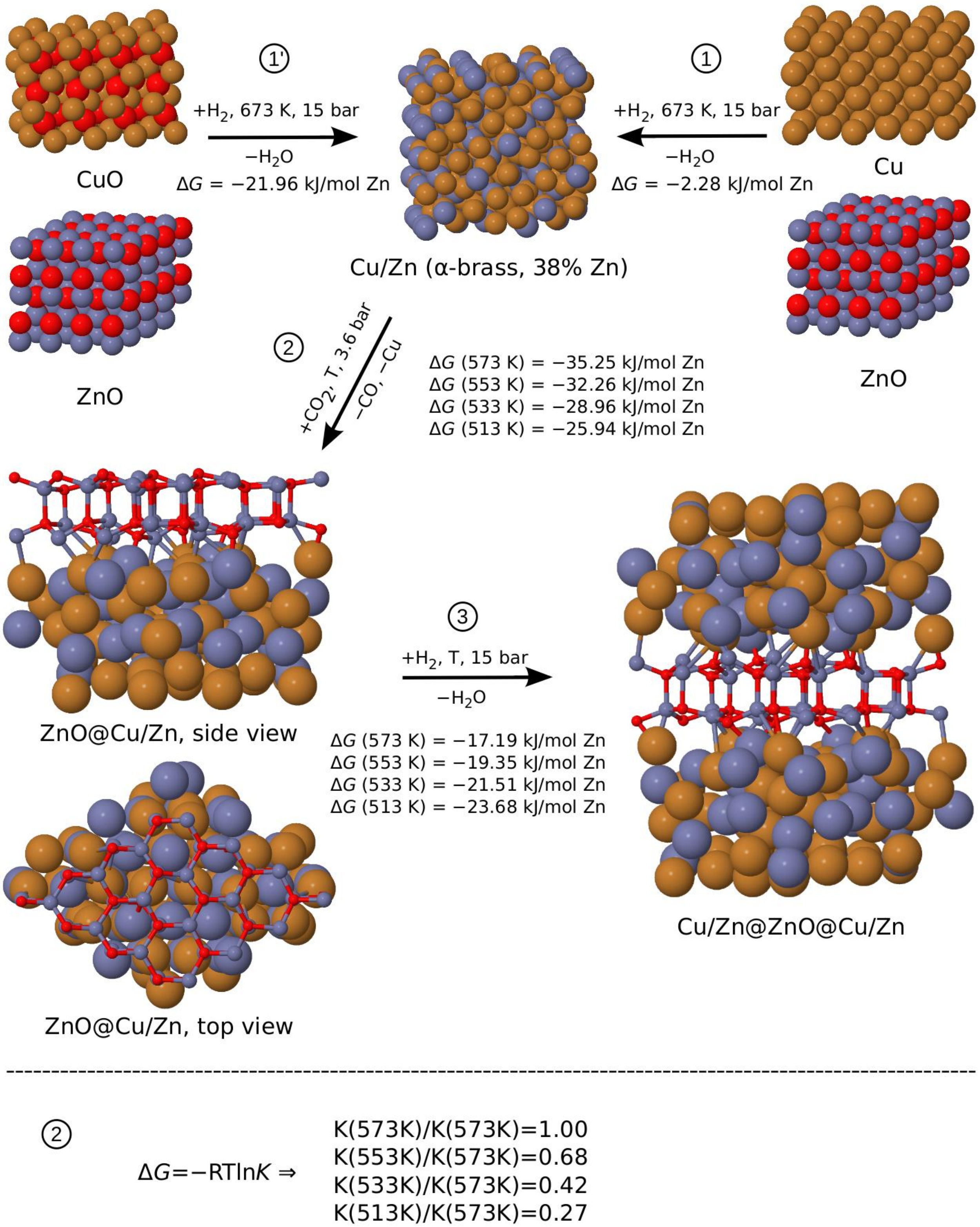

Supplementary Fig. 17. Schematic representation of the thermodynamics of the formation of alloy (Equation 2), the ZnO layer (Equation 3), and the alloy on the layer of ZnO (Equation 4).

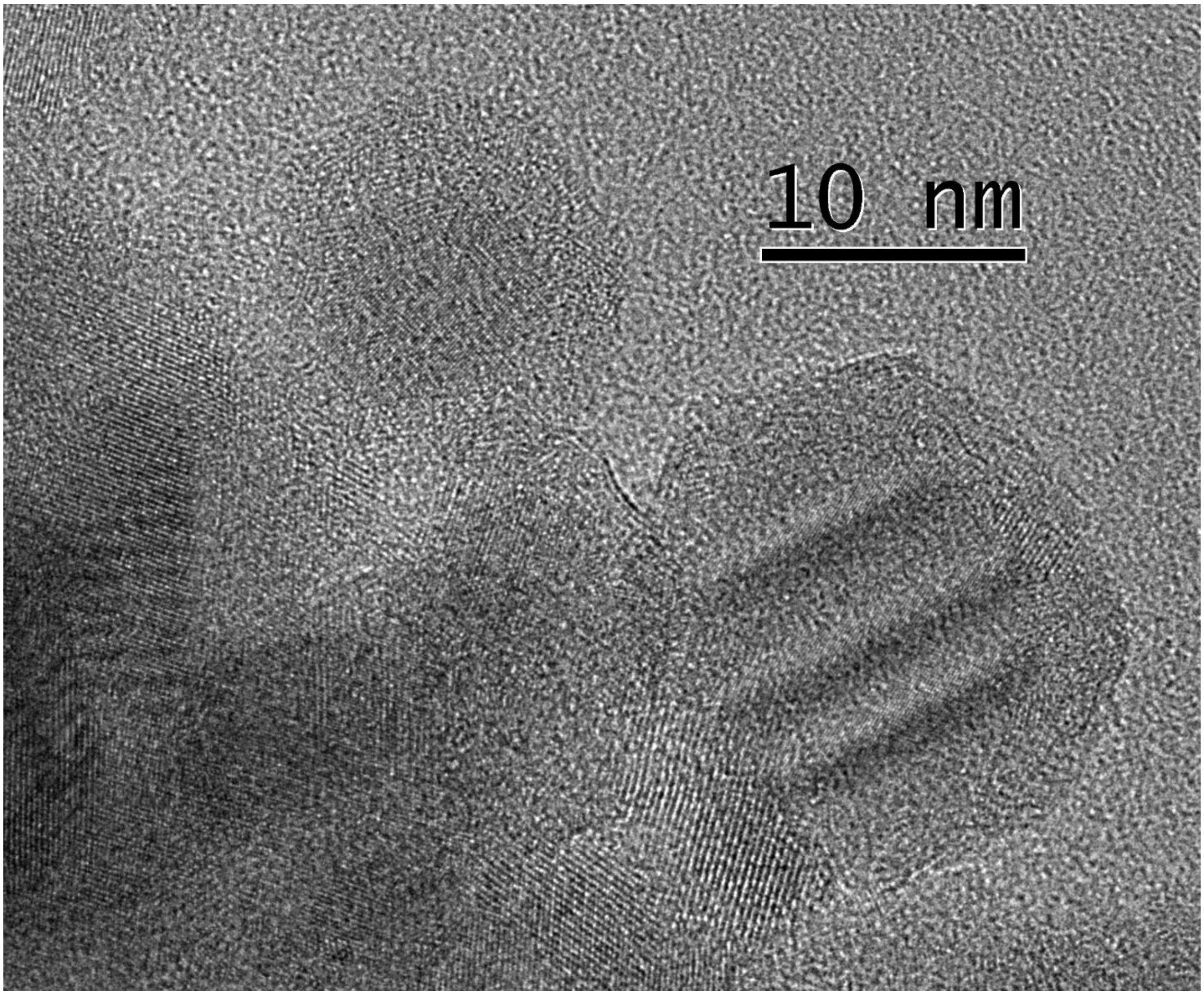

Supplementary Fig. 18. HRTEM micrograph of CZA catalyst after pre-treatment at 673 K and 15 bar of hydrogen and switching to CO_2/H_2 reaction mixture at 533 K.

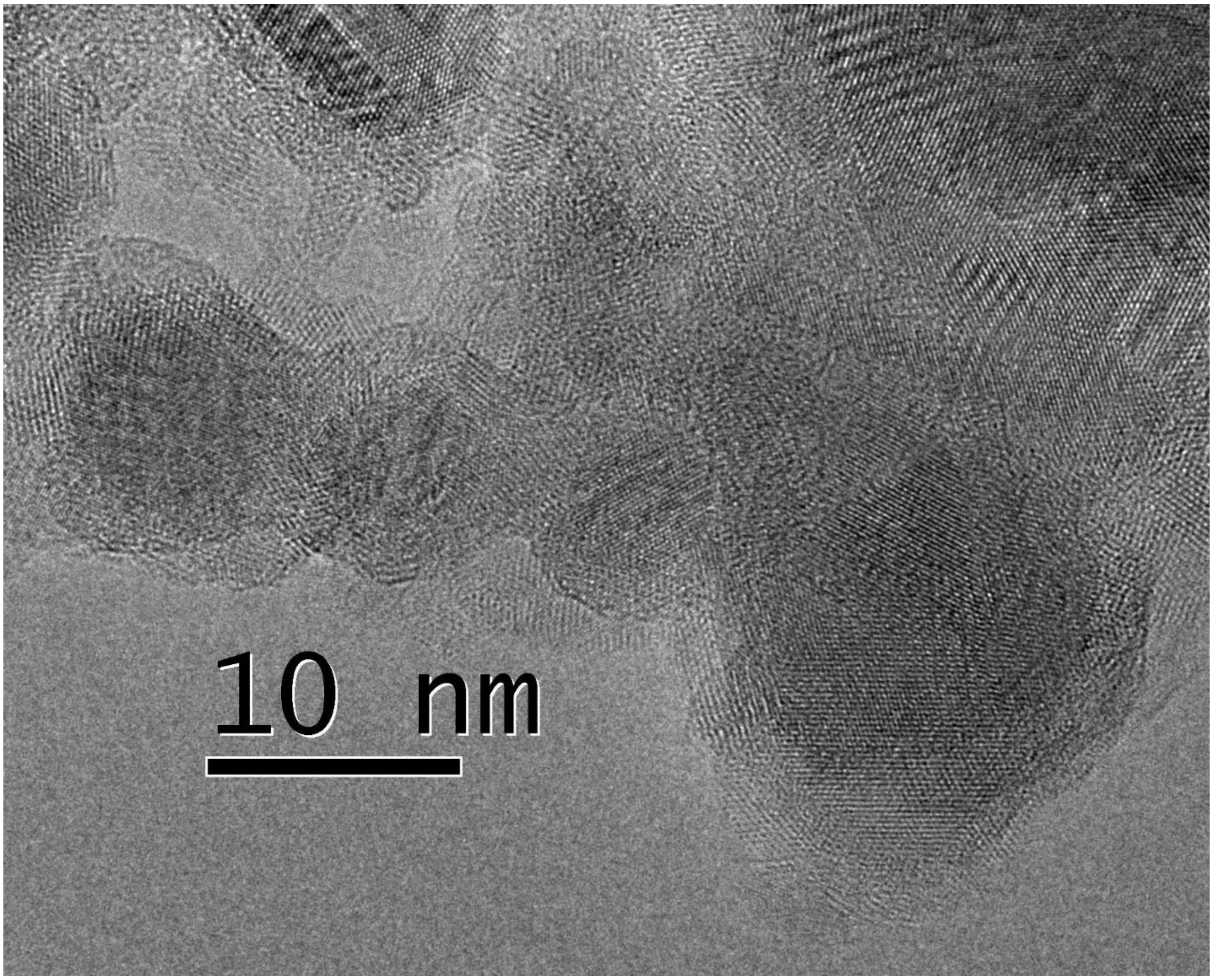

Supplementary Fig. 19. HRTEM micrograph of CZA catalyst after pre-treatment at 673 K and 15 bar of hydrogen and switching to CO_2/H_2 reaction mixture at 533 K.

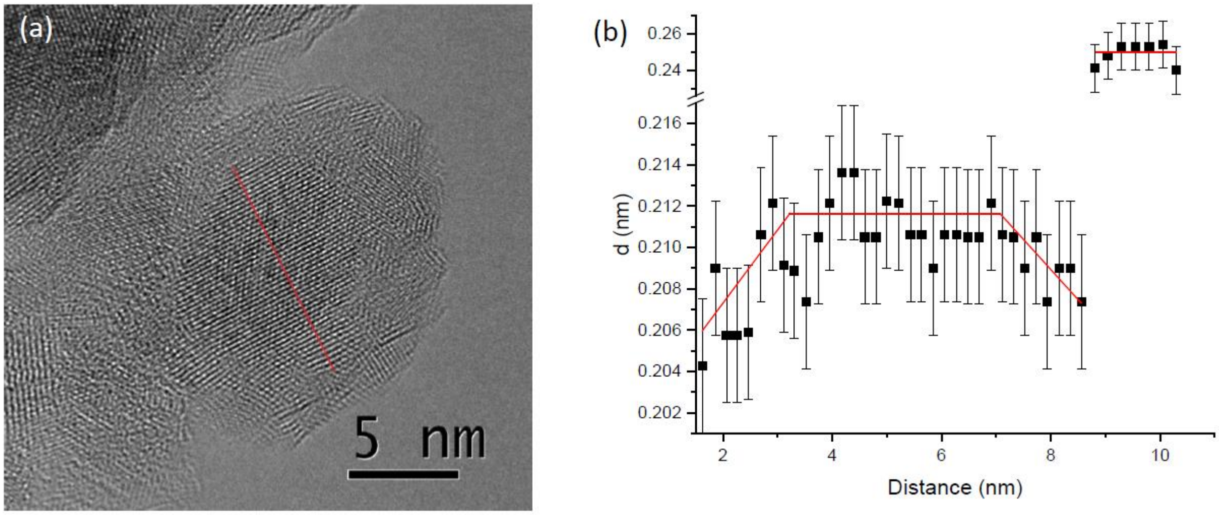

Supplementary Fig. 20. (a) HRTEM micrograph of CZA catalyst after pre-treatment at 673 K and 15 bar of hydrogen and switching to CO_2/H_2 reaction mixture at 533 K (red line indicates line-profile for d spacing analysis); (b) detailed analysis of d spacing profile (error bars represent SD, red lines are shown for better visibility only).

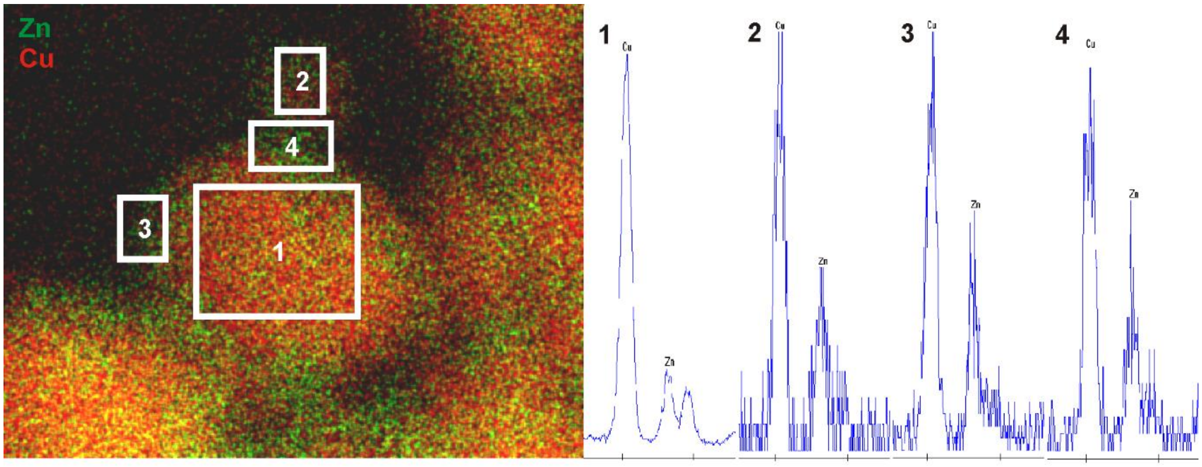

Supplementary Fig. 21. EDX mapping of CZA catalyst pre-treated at 673 K and 15 bar in hydrogen and after that reacted with CO_2/H_2 reaction mixture at 533 K and 15 bar for 3 hours, as well as extracted EDX spectra for different region marked on the EDX map.

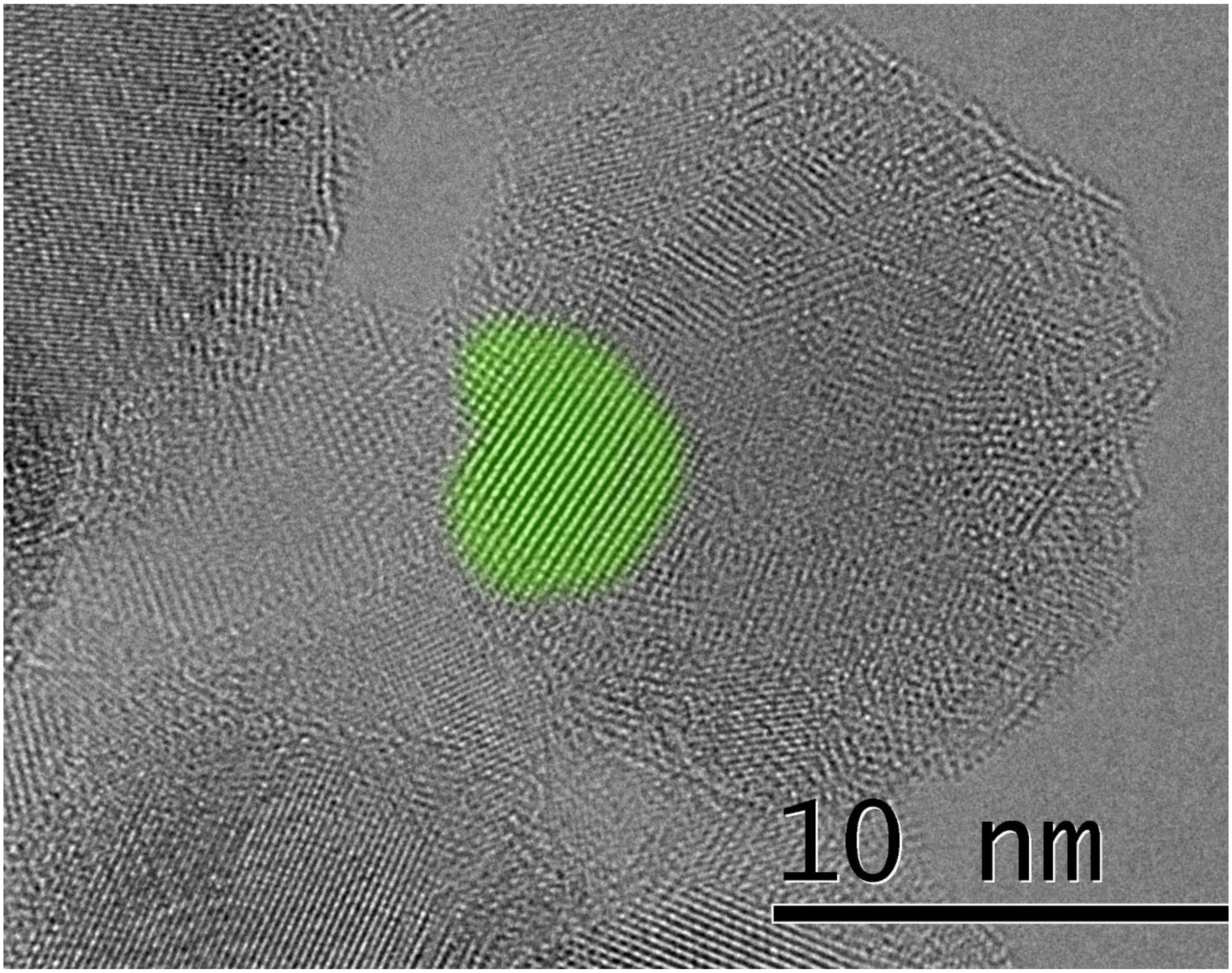

Supplementary Fig. 22. HRTEM micrograph of CZA catalyst pretreated in H_2 at 673 K and re-oxidized with the CO_2/H_2 mixture at 573K. Green mask highlights zinc oxide nanoparticle with an interplanar spacing of 0.26 nm, which corresponds to (002) planes of zinc oxide.

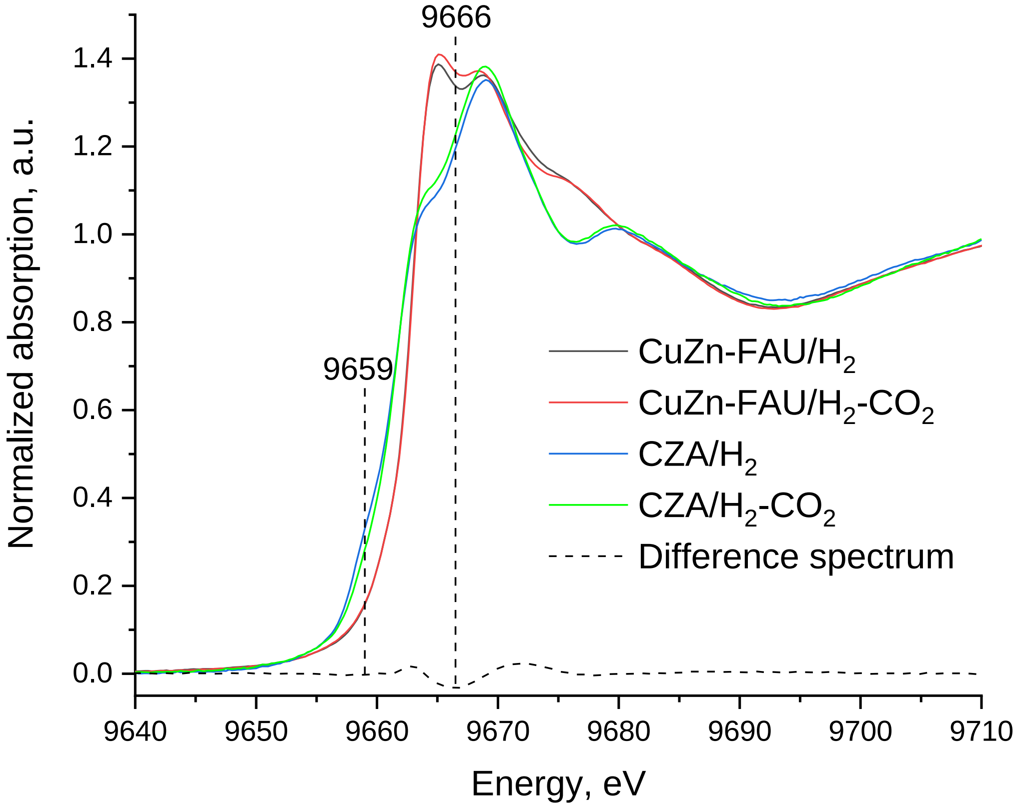

Supplementary Fig. 23. Zn K-edge XANES spectra of CuZn-FAU and CZA catalysts under operando conditions of carbon dioxide hydrogenation (H_2-CO_2 mixture with ratio 3:1, 15 bar and 533 K) and after switch to pure hydrogen (15 bar, 533 K), as well as difference spectra obtained by subtraction of the spectrum of CuZn-FAU sample collected in CO_2/H_2 atmosphere from the one of the same sample under hydrogen.

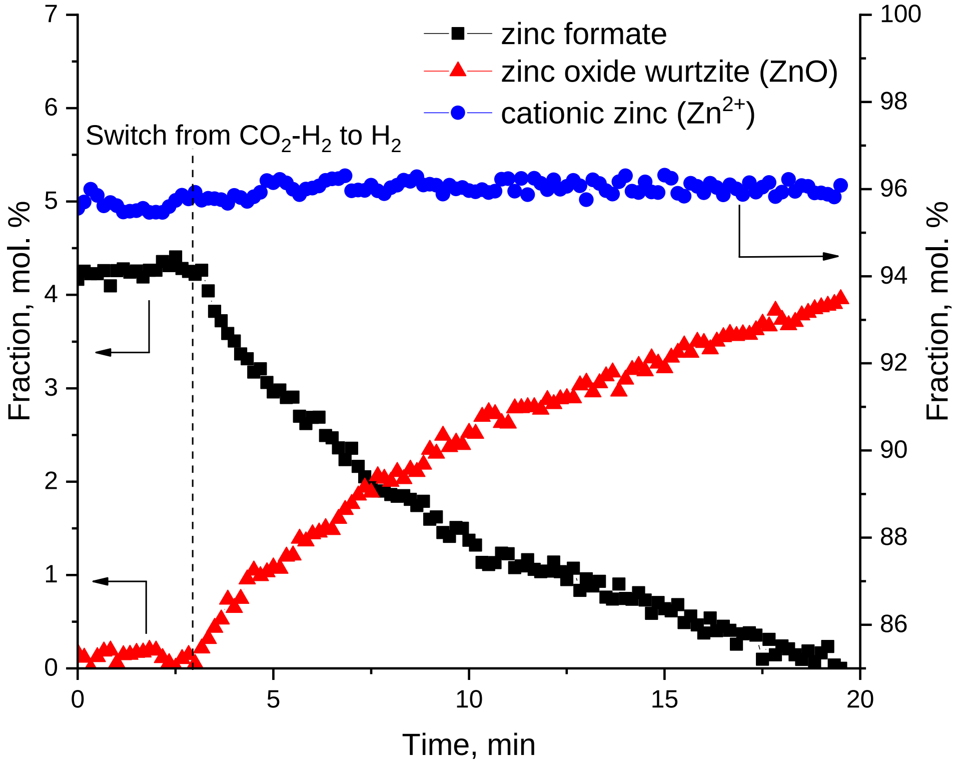

Supplementary Fig. 24. The relative fractions of zinc oxide wurtzite, zinc formate and cationic zinc determined from PCA analysis of XANES spectra as function of time after switch from CO_2/H_2 mixture to hydrogen at 533 K and 15 bar over CuZn-FAU catalyst.

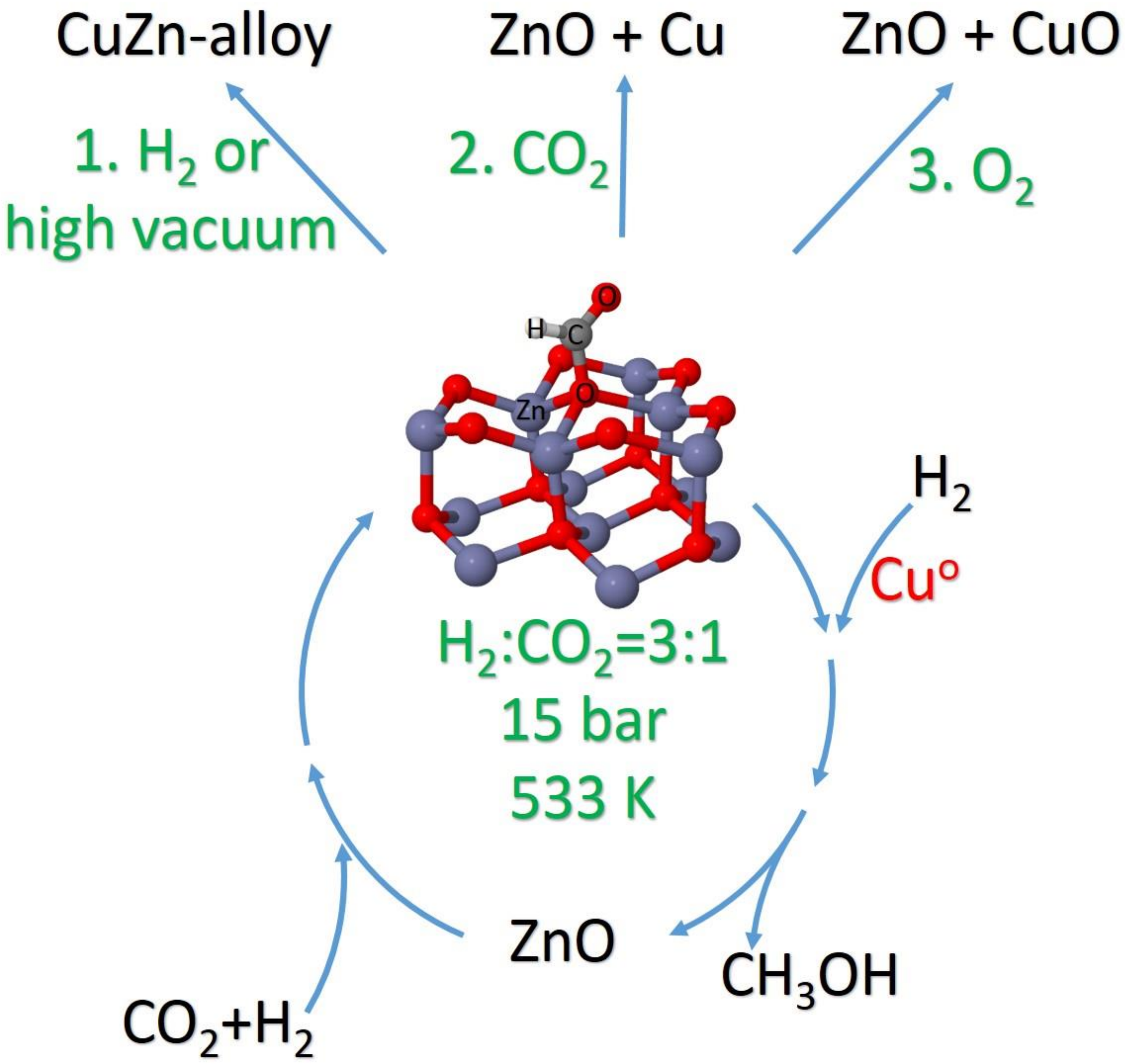

Supplementary Fig. 25. Catalytic scheme of carbon dioxide hydrogenation highlighting zinc formate as reactive intermediate as well as other often-suggested structures and their possible role in the catalytic cycle (copper-zinc alloy; zinc oxide and copper or zinc oxide and copper oxide) which can be observed under different, non-catalytic conditions, such as high vacuum, highly reducing and oxidizing conditions.

Supplementary Table 1. Results of oxygen chemisorption test, PCA analysis of Zn K-edge XANES as well as methanol selectivity and productivity during carbon dioxide hydrogenation experiment (533 K and 15 bar) for CZA catalyst under different pre-treatment conditions.

Pretreatment conditions	Methanol production, mmol g^{-1} min^{-1}	Methanol selectivity, %	Reduced zinc, mol. %	$S_{(Cu)}$, m^2 g^{-1}
673 K and 15 bar in H_2	0.38±0.02	54±2	49±1	41±3
533 K and 15 bar in H_2	0.44±0.02	43±2	16±1	52±3
673 K and 15 bar in H_2; after that 15 bar in CO_2/H_2 mixture at 573K	0.28±0.02	48±2	33±1	29±3

Supplementary Table 2. Comparison of amount of zinc in copper-zinc alloy for CZA catalyst at different reaction conditions determined either by using Vegard's law and position of (220) diffraction peak or PCA analysis of Zn K-edge XANES. Data was collected at SNBL BM31 beamline, ESRF, France.

Pre-treatment	Conditions during measurement	Amount of reduced zinc based on PCA analysis, mol. %	Position of (220) diffraction peak, °	d(220) at 533K, nm	Amount of zinc in CuZn alloy based on PCA analysis and assuming that all reduced zinc is present in the form of copper-zinc alloy, mol. %	Amount of zinc in CuZn alloy calculated by Vegard's law based on position of (220) diffraction peak at 533K, mol. %
Reduced in hydrogen at 533 K and 15 bar	As-pretreated	26.3	22.199	0.12921	9.2	7.7
	After switch to CO_2/H_2 mixture	15	22.223	0.12907	5.5	6.0
	After back switch to hydrogen	22.7	22.201	0.12925	8.0	8.3
Reduced in hydrogen at 573 K and 15 bar	As-pretreated	36.6	22.162	0.12943	12.3	10.6
	After switch to CO_2/H_2 mixture	16.5	22.205	0.12918	6.0	7.4
	After back switch to hydrogen	23.7	22.186	0.12929	8.4	8.8
Reduced in hydrogen at 673 K and 15 bar	As-pretreated	56.6	22.070	0.12996	17.9	17.4
	After switch to CO_2/H_2 mixture at 15 bar and 573K	33	22.160	0.12944	11.3	10.7

Supplementary Table 3. Thermal effect of the hydrogen adsorption over Cu(111) and CuZn(111) surfaces.

Model	ΔE, kJ·mol^{-1}
H_2@Cu(111)	–90.7
H_2@Cu/Zn(111)	–9.6

Supplementary Table 4. Results of catalytic carbon dioxide hydrogenation experiment over CZA and CuZn-FAU catalysts measured at 533 K, 15 bar and H_2/CO_2 molar ratio of 3.

Sample	Methanol production, mmol g^{-1} min^{-1}	Methanol selectivity, %	Normalized methanol production, mmol g_{Cu}^{-1} min^{-1}
CZA catalyst pretreated at 673 K and 15 bar in H_2; after that 15 bar in CO_2/H_2 mixture at 573K	0.28±0.02	48±2	0.55±0.04
CuZn-FAU	0.011±0.001	40±2	0.42±0.04

Supplementary Note 1

Zn *K*-edge XANES: temperature programmed reduction (TPR), principal component analysis (PCA), and comparison with Zn *K*-edge EXAFS

The normalized Zn K-edge X-ray absorption near edge structure (XANES) spectrum of the CZA catalyst pretreated in a flow of helium shows a main peak located at 9669.2 eV with a shoulder at 9664.0 eV, which is indicative of the dominant presence of the wurtzite zinc oxide phase [1]. After the activation in hydrogen, a characteristic pre-edge shoulder at 9659 eV appeared in the Zn K-edge spectrum (Supplementary Fig. 2a). The assignment of this peak to copper-zinc alloy was made on the basis of a H_2-TPR-XAS experiment. TPR of the catalyst was performed under both 1 bar of flowing H_2 and under flowing H_2 at 15 bar pressure. Supplementary Fig. 2b shows the evolution of the Zn *K*-edge during TPR under H_2 at 1 bar to 773 K. These TPR data were then submitted to PCA analysis to establish the spectroscopic character and thermal evolution characteristics of the Zn species formed and consumed.

Supplementary Fig. 3a shows the nature of the two Zn components (red) found to be present during TPR using PCA. These are compared to standard spectra (black) derived from ZnO (Wurtzite) and $Cu_{0.67}Zn_{0.33}$ (alpha-brass) both measured at ambient temperature. Supplementary Fig. 3b shows an examples of the quality of the reproduction of the experimental data by PCA analysis for two different extents of Zn reduction (as indicated). Black lines = experiment; red lines = PCA calculated spectra; blue lines = residuals. Supplementary Fig. 3c shows the temperature dependence of the formation of reduced Zn (Zn^0) for the two cases investigated: black symbols = 15 bar H_2; red symbols = 1 bar H_2.

These data show, that the formation of reduced Zn, and therefore a supported Cu_xZn_y phase, is significantly dependent upon the pressure of hydrogen. This is important as it clearly demonstrates that the nature of the catalyst that may be obtained, in and around the thermal regime used for selective methanol synthesis catalysis, is a significant function of the H_2 pressure applied. A catalyst reduced at ambient pressure to the methanol synthesis reaction temperature (533 K) is shown by this measure to contain ca. 4.5-5 times (0.35/0.075) less Zn^0 than the same catalyst reacted under 15 bar H_2. To achieve the same level of Zn reduction under 1 bar H_2 then a reduction temperature of temperature of 680 K would have to be employed.

Supplementary Fig. 4 (a) compares the evolution of the Zn K edge EXAFS (as Fourier transforms of the k^3-weighted EXAFS) with (b) the PCA derived levels of Zn reduction achieved for reduction achieved under 15 bar H_2 (left hand axis) and the shell integral (indicated by the blue shaded area in Supplementary Fig. 4a corresponding to the scattering feature in the FT (right hand axis).

The XANES/PCA analysis indicates that at the reaction temperature of 533 K, and under 15 bar of H_2, ca. 35 % of the Zn is reduced. Given the Cu:Zn stoichiometry of the sample ($Cu_{2.6}Zn$) this means that, if all of the reduced Zn is contained within an *fcc* (alpha) brass phase then the maximum stoichiometry that this phase could have achieved would be ca $Cu_{7-7.5}Zn$.

However, comparison of the XANES with the Fourier transform of the k^3-weighted Zn *K*-edge EXAFS, shows that there is an appreciable range of temperatures (ca. 370 – 500 K), wherein Zn is being reduced (up to ca. 25 % Zn^0), yet no evidence is found for the presence of a significant

CuZn scattering interaction (we note that by 578 K (ca. 49 % Zn^0) such a feature is clearly present in the EXAFS).

This would suggest firstly, that evidence of Zn reduction as indicated by the Zn *K*-edge XANES does not necessarily correspond to the formation of a copper-zinc alloy phase; and secondly, that the formation of an alpha brass phase to ca. 25 % of the total Zn present should be detectable in EXAFS. Instead we might postulate that the first, low temperature, stage in the formation of the alpha-brass phase may well be that of the formation of Zn point defects. These point defects accumulate before they are eventually transformed into a growing copper-zinc alpha-brass phase with the concomitant appearance of a clear CuZn scattering feature in the EXAFS.

If this is the case then the value returned from analysis of the XANES regarding the levels of reduced zinc present at the reaction temperature of 573 K, and therefore the degree of copper-zinc alloy formation indicated from the XANES, must be regard as very much an upper limit. The totality of the reduced Zn could in fact represent sum of the population of such defects and that zinc that is incorporated into the copper-zinc alloy.

On this basis we should state that the XANES and the Zn K-edge EXAFS combined would conclude that at 533 K and under 15 bar H_2 then the average alpha-brass phase formed prior to reaction with H_2/CO_2 can be no more than, and most likely somewhat less than, Cu_7Zn. (see Supplementary Table 2).

Zn K –edge XANES during reactive switching at elevated pressure: principal component analysis (PCA)

Supplementary Fig. 5 shows representative data obtained from PCA analysis of Zn K-edge XANES made during the switch from 15 bar H_2 to 15 bar CO_2/H_2 mixture giving exemplars of the quality of spectral reproduction of the individual components found by PCA and how they compare to known standard materials.

These data show that the PCA analysis not only accurately reproduces the experimental spectra but also that the component spectra generated from this transient experiment can be used to specify the speciation present with a high degree of precision. Most pertinently, component 3 is shown to have a spectral character very close to that derived from a solid zinc formate sample and shows a character that is distinct in a number of ways from that which might be expected from, for instance, a carbonate like species. In this sense, the PCA can be seen as consistent with the SSITKA-infrared experiments that identify the reactive species as a formate rather than a carbonate. More importantly, however, the PCA-XANES identifies formate associated with the zinc as a reactive species.

Operando, steady state, Zn *K*-edge EXAFS

Supplementary Fig. 6a gives examples of Zn *K*-edge, k^3-weighted EXAFS (black), collected in situ and under steady state conditions, for four different points within an activation and then cycling between 15 bar H_2 and 15 bar of the H_2/CO_2 catalytic feedstock. The EXAFS from a bulk

ZnO (wurtzite) standard is also given. Supplementary Fig. 6b gives the corresponding Fourier transforms of the k^3-weighted data.

The Zn *K*–edge EXAFS, measured before and after activation, and then in operando under successive switches between 15 bar H_2 and then 15 bar CO_2/H_2 feeds, reveals evidence of a considerable change in the habit of the phases present in response to the changing of the feedstock that goes beyond the redox behavior already observed and delineated using XANES.

Reduction of the sample, under 15 bar of H_2 to 533 K, as loaded precipitates the removal of much of the ZnO phase initially present. Concomitantly, two new features appear between ca 2.4 and 3 Å in the Fourier transform. The first of these is that of a Cu-Zn interaction that is expected if a significant portion of the Zn has been reduced and incorporated into the Cu phase to yield a the copper-zinc alpha brass.

The second feature, however, cannot be explained on this basis. Attempts to fit this new shell using a second, longer, CuZn interaction or a second, shorter Zn-Zn scattering path result in physically unacceptable Debye-Waller factors in each case. Instead this feature may be satisfactorily fitted through the addition of a new Zn-Al scattering contribution.

The appearance, or rather increased contribution of this feature to the Zn *K*-edge EXAFS, can be resolved most easily as a result of the thinning/depletion of the ZnO phase that must accompany the extraction of Zn from the ZnO as it is incorporated into the nascent copper-zinc alpha-brass phase. As this occurs the contribution from that Zn that exists at the interface between the ZnO and the Al_2O_3 support becomes more significant from the perspective of the Zn *K*-edge EXAFS.

It may also be the case that a loss of a significant proportion of the zinc from the ZnO phase to the copper zinc alloy might also trigger a morphological rearrangement of that oxidized zinc that remains, such that it wets the alumina in order to maximize the ZnO/Al_2O_3 interface. This, however, must remain only a speculative possibility on the basis of this EXAFS data.

After switching the feed to a catalytic mixture of CO_2 and H_2 at 15 bar, and allowing a steady state to develop, it is observed that both the scattering due to the formation of the copper-zinc alloy and that due to the Zn-Al interaction diminish greatly, whilst the first shell Zn-O scattering grows in intensity. These changes are entirely consistent with the change in the reaction mixture eliciting a partial, but significant, re-oxidation, of the Zn and the movement of this fraction of re-oxidized material back into the ZnO phase at the expense of the copper-zinc alloy. The relative diminution of the ZnAl scattering feature that accompanies this suggests a concomitant re-growth of the ZnO phase.

Operando Cu K-edge EXAFS and XANES at steady state

Supplementary Fig. 7a shows the k^3-weighted Cu *K*–edge EXAFS derived from the sample in three cases (as indicated) along with that derived from both Cu and $Cu_{0.67}Zn_{0.33}$ foil standards at ambient temperature. Supplementary Fig. 7b shows the corresponding phase corrected Fourier transform representations of the k^3-weighted data.

Copper and zinc, lying next to each other in the periodic table are, to all intents and purposes indistinguishable from the viewpoint of the Cu *K*-edge EXAFS. The structure of the copper-zinc

alphas-brass is also fcc, as is that of pure copper. As such, distinguishing the presence of zinc in the copper structure is only a priori achievable on the basis of the phase shift (most clearly evident in the k^3-weighted EXAFS (*) that results from the expansion of the *fcc* lattice parameter as a function the amount of incorporated zinc and/or the increased disorder in the system that the zinc induces. This latter effect is most apparent in the amplitudes of the k^3 EXAFS but also is evident from the diminution of the higher fcc shells highlighted in blue in Supplementary Fig. 7b. It is also the case that EXAFS derived from copper and copper zinc mixtures is very sensitive to thermal affects. Quantitative analysis therefore requires asymmetric contributions to the pair-distribution function, that affect both the amplitude and the apparent phase of the EXAFS, to be accounted for even in the case of the bulk standard cases [2,3].

Comparing the spectra derived from the standards and the catalyst reduced at 15 bar H_2 at 673 K and measured in H_2 at 533 K we see that this challenging situation is further complicated in the case of the real catalyst.

However, even in a challenging situation such as this some salient observations can be made from changes in operando Cu K-edge EXAFS that can be seen to be consistent with the formation and then removal of a copper-zinc alloy phase as a function of reactive environment.

From the Cu K-edge data obtained for the catalysts reduced at 673 K and maintained at 533 K under 15 bar H_2, and the subsequently measured again under a reaction mixture comprised of both CO_2 and H_2 two significant changes are evident. Firstly, under the reaction mixture we observe a ca. 15 % increase in the intensity (shell integral) of the first scattering shell and a concomitant change in both the overall and relative intensities of the higher fcc shell structure.

By reference to the two standard foils we can observe that similar differences exist between the pure copper foil and that copper-zinc brass. Irrespective of the temperature therefore, such changes would be entirely consistent with the removal of Zn from a CuZn phase occurring as a result of the change in the reactive feedstock. The relative changes that occur within the higher shell structure might also be indicative of changes in the morphology of the copper particles that result from alloying/de-alloying [4]. However, on the basis of this data alone, and as with the possibility of reduction induced wetting of the Al_2O_3 phase by that zinc that remains oxidized, this must remain a speculative proposition.

Detailed analysis of Cu K-edge XANES spectra (Supplementary Fig. 8) during the gas transient switch from hydrogen to CO_2/H_2 reaction mixture revealed the lack of any oxidation of copper and it confirms the absence of the formate intermediate on the copper surface. These spectra correspond to the nanoparticulate metallic copper phase only, and changes after transient switch to CO_2/H_2 reaction mixture (see black difference spectrum in Supplementary Fig. 9) revealed only the process of partial de-alloying of copper-zinc alloy which was already discussed in Cu K-edge EXAFS section (Supplementary Fig. 7). As can be seen, difference spectra between CZA catalyst acquired in hydrogen atmosphere and the same catalyst after transient switch to CO_2/H_2 reaction mixture (black line, Supplementary Fig. 9) corresponds well to the difference spectrum of copper foil and copper-zinc foil standards (red line, Supplementary Fig. 9).

Despite that the dispersion of copper in commercial CZA catalyst accordingly to results of chemisorption experiment is 16 % (d_{Cu}≈ 7 nm; 1/6 of total copper atoms represent surface atoms), such quantity of surface atoms is sufficiently high to detect any possible changes taking place on the catalyst surface during catalytic cycles by such bulk technique as XAS. To strengthen the argument, we have also investigated a sample containing 1 wt. % of copper on zinc oxide with very high dispersion of Cu (≈ 1-2 nm according to EXAFS fitting) with the help of operando XAS in order to probe any possible changes taking place on the copper surface during a transient switch from hydrogen to CO_2/H_2 reaction mixture at 15 bar and 533 K. Even for such highly dispersed copper nanoparticles, there is no evidence of surface or bulk oxidation due to forming of formate species. The position of the white-line remains constant, and the difference spectrum (blue curve, Supplementary Fig. 10) represents only noise in the region 8975-9000 eV.

Investigation of possible oxidation of Cu surfaces under pure carbon dioxide

In order to test possibility of a copper oxidation by carbon dioxide, qXAS data was acquired during transient switch from CO_2/H_2 reaction mixture to carbon dioxide at 15 bar and 533 K. As it was already discussed (Supplementary Fig. 8), under methanol synthesis conditions copper is present in metallic form without any evidence of cationic copper species. Supplementary Fig. 11 shows that the switch to carbon dioxide does not result in oxidation of copper even under pure carbon dioxide.

Supplementary Note 2

Additional IR observations during the switch from CO_2/H_2 gas reaction mixture to pure hydrogen

Infrared spectroscopy plays an important role in studying the hydrogenation of carbon dioxide to methanol over copper-containing catalysts. Many groups significantly contributed to this field, reporting the analysis of IR spectra taken for different catalysts and under different reaction conditions, in particular, temperature and pressure. While the bulk assignment of the species, such as formate and methoxy, observed in infrared spectra does not create a confusion anymore, a more detailed analysis attempting to identify the localization of the surface species is heavily debated. It is, however, highly desirable, since it would enable the direct observation of active and spectator species.

Starting from the first works by Fujita et al. [5,6] the existence of two types of formate species, formed from carbon dioxide over copper-zinc catalyst, has been suggested. The first type, as suggested by the authors, is represented by the formate species located over copper sites and characterized by the set of bands at 2930, 2850, 1620 and 1350 cm^{-1}. The second type corresponds to the zinc-formate species, giving the IR bands at 2970, 2880, 2740, 1580, 1383 and 1365 cm^{-1}, as assumed by the authors. Notably, the assignment of the bands due to copper-formate was done by correlating the experimentally observed spectrum with the literature data of copper-formate formation detected by EELS on the surface of Cu(100) crystal. Moreover, the bands due to the copper-formate detected by EELS have a significant offset with respect to the bands detected over Cu/Zn catalyst. Authors themselves admit that the position of the formate band is very sensitive to the composition of the catalyst and can drift significantly.

From the copper-formate reactivity point of view, Fujita et al. [6] make an important conclusion that copper-formate does not fully convert to methanol upon reacting in a flow of hydrogen: the amount of methoxy species observed was 5 times lower than expected. Apparently, a significant fraction of copper-formate undergoes decomposition instead of hydrogenation to methanol. This observation was comprehensively studied by the group of Campbel, who concluded that copper-formate species can not be the precursor of methanol [7–10]. Using the model catalysts that are unsupported metallic copper and copper deposited over silica, they showed that the rates of formate decomposition does not correlate with the rate of methanol formation and the difference is dramatic. Quantitative titration revealed that no more than 3% of copper-formate species undergo the conversion to methanol [9]. Measurements of the apparent activation energies and kinetic isotope effect over these catalysts confirmed the non-formation of methanol from copper-formate.

The copper supported over silica catalyst was widely used for studying the copper-formate role in the production of methanol from carbon dioxide. Numerous IR studies showed that the position of the bands due to the copper-formate is strongly affected by the nature of the catalyst and the pretreatment history. Hence, the formate species formed during the reaction of carbon dioxide with hydrogen are often located at higher frequency about 1590 cm^{-1} with respect to the formate species obtained by the adsorption and followed evacuation at high temperature of formic acid (~1550 cm-

[1]) [11]. Similar behavior can be observed when the surface of the catalyst is partially oxidized by flowing oxygen after the reaction (shift from 1550 to 1574 cm^{-1} [10]). This sensitivity of the copper-formate band position to the redox potential of the feed and the pretreatment conditions makes the assignment of the IR bands by the frequency challenging.
Together with the pretreatment conditions, the reaction conditions themselves are important and affect the resulting activity and IR spectra. Notably, all aforementioned studies were performed under considerably different conditions: the temperature varied from 353 to 523 K, the pressure varied from atmospheric to 6 bar, H_2/CO_2 ratio varied from 3 to 9. Obviously, this variation leads to the effectively different composition of the surface of the catalysts under working conditions, which in turn can favor the stabilization of different types of surface species, including formates. This is extremely important to bare this fundamental issue in mind and treat it carefully, since the composition of the active sites of the catalyst is a function of the composition of reacting media. Therefore, the methods that do not induce any changes in the steady-state operation of the catalyst are highly desirable for the unambiguous determination of active surface species. In designing our experimental work, we followed this approach and used the steady-state IR spectroscopy coupled with isotope transient analysis (see Fig. 1 and Supplementary Fig. 1). Furthermore, non steady-state approach similar to that previously published in the literature was tested as well; however, the result of this experiment should be discussed only together with SSITKA-FTIR findings (Supplementary Fig. 12).

Non-steady state switch from hydrogen to CO_2/H_2 reaction mixture over CZA catalyst results in a rapid development of the main band at 1597 cm^{-1} with a broad shoulder, which is centered at 1510-1520 cm^{-1}. Similarly, the switch back to pure hydrogen leads to the slow disappearance of both the main peak and the shoulder. Both bands can be assigned to formate species, which is in line with the literature data. Formates band in the region 1585-1593 cm^{-1} was previously attributed to the formate species localized on zinc oxide [12,13]. During our operando XAS experiment conducted under the same conditions as non-steady-state FTIR switches, we observed the formation (during transient switch from hydrogen to CO_2/H_2 mixture) and decomposition (during the back switch from CO_2/H_2 to hydrogen) of zinc formate (Fig. 2). The simultaneously detected MS-response (Supplementary Fig. 13) indicates that, the main products during decomposition of the formate species under the hydrogen atmosphere are methanol and carbon monoxide. Comparison of zinc formate decay response (determined by XAS) with the cumulative methanol yield (integral of MS-response of methanol) multiplied by (-1), confirms that kinetic of methanol evolution follows well the zinc formate decomposition trend (Supplementary Fig. 14). Based on this experiments and the previous work of Fujitani et al. [13] and Kähler et al.[12], we have attributed the main peak at 1597 cm^{-1} to formate species localized on ZnO. The position of the shoulder at 1510-1520 cm^{-1} might indicate its assignment to the copper-formate, discussed above, due to the high lability of the position of this band. However, taking into account the results of operando XAS examination, where we did not observe any changes in the oxidation state of copper, such interpretation remains questionable. Despite of the presence of the band, which might resemble the band due to the copper-formate, its involvement in the catalytic cycle requires further analysis. By comparing the

IR spectra acquired during the steady-state switches (Supplementary Fig. 1), achieved by changing the isotope composition ($^{12}CO_2$ to $^{13}CO_2$) of the feed and non-steady-state switches (Supplementary Fig. 12) by changing CO_2/H_2 to pure hydrogen, we can clearly notice the difference in behavior of the formates localized at 1510-1520 cm^{-1}.

The analysis of the spectra obtained during the steady $^{12}CO_2/H_2$ to $^{13}CO_2/H_2$ isotope switch reveals that the broad shoulder at 1510-1520 cm^{-1} is still there, but does not change its position and does not participate in the isotope exchange (Supplementary Fig. 1). This is also visible from the Fig. 1 in the main text. This experimental observation points to the possible presence of a small fraction of copper-formate, which is, however, not active in the methanol synthesis, since it does not respond upon isotope switch. This shoulder does not participate in isotope exchange during SSITKA experiment (Fig. 1) and therefore these species, regardless of the exact assignment, are spectators in the methanol synthesis reaction.

Apparently, the high stability of the species, characterized by the band at 1510-1520 cm^{-1} during the steady-state isotope switch, can be explained by the significantly different redox potential of pure hydrogen with respect to CO_2/H_2 mixture. The highly reducing hydrogen modifies the surface of CZA catalysts, making the decomposition of the formate species characterized by the 1510 cm^{-1} bands favored. Equally, however, they are stable under the CO_2/H_2 environment, as evidenced from the $^{12}CO_2/H_2$ to $^{13}CO_2/H_2$ isotope switch. Perhaps, such a high stability of these species under CO_2/H_2 environment makes isotope exchange, and therefore, further hydrogenation to yield methanol, significantly hampered.

Another difference between the steady-state and not-steady-state switches is significantly different kinetics observed during the transient SSITKA switch and the back switch from CO_2/H_2 gas reaction mixture to hydrogen (Fig. 2c and Supplementary Fig. 12). Such a slow kinetic behavior during decomposition of formate species in pure hydrogen compared to SSITKA is pointing out that the catalyst surface is significantly different compared to the working catalyst surface during the steady state catalyst operation, and can be attributed to a rearrangement of the catalyst structure after the switch from CO_2/H_2 gas reaction mixture to pure hydrogen. According to the time resolved operando XAS experiment, we observed immediate formation of copper-zinc alloy after the transient switch from CO_2/H_2 gas reaction mixture to pure hydrogen. Therefore, working copper-zinc oxide interface undergoes changes and the formation of copper-zinc alloy occurs on the catalyst surface. Theoretical calculation (Supplementary Table 3) predicts that the enthalpy of hydrogen chemisorption over copper-zinc alloy surface is only 9.6 kJ mol^{-1}, making the activation of hydrogen highly unlikely, while pure Cu (111) can activate and split molecular H_2 efficiently. This is in line with a slow formate hydrogenation kinetics observed during the transient switch to pure hydrogen compared to SSITKA experiment (Fig. 2c), since in the H_2 atmosphere the zinc oxide phase undergoes reduction to copper-zinc alloy, which inhibits the rate of the formate hydrogenation step.

In conclusion, our IR results can not exclude the presence of a small fraction of copper-formate on the surface of the working catalyst. However, these species are not isotopically labile and, therefore, do not participate in the methanol synthesis reaction. The discrepancy with previous

literature data most probably arises from the considerable dispersion in the reaction conditions used before, such as temperature and pressure, which apparently leads to the different structure of the surface of the working catalyst. Furthermore, non-steady-state and even non operando approaches widely used in the previous works cannot act as a basis for any unambiguous conclusions regarding the participation of any species in the catalytic cycle. As such, possible presence of small fraction of copper-formate as shown in Supplementary Fig. 12 and other works [5,6], does not necessarily mean participation of these species in the catalytic cycle as was further confirmed by SSITKA-FTIR experiment (Fig. 1 and Supplementary Fig. 1).

Supplementary Note 3

XAS investigation of steady-state catalyst operation after the transient switch from pure hydrogen to CO_2/H_2 gas reaction mixture

The fact that CZA catalyst is stable over the timescale of minutes during the transient switches reported in the main text (Fig. 2) does not make it safe to conclude that a steady state operation was reached and the catalyst is not affected by the pretreatment and/or transient switches. In order to investigate this issue, we have conducted additional XAS experiment (Supplementary Fig. 15), where we have monitored CZA catalyst after the transient switch from pure hydrogen to CO_2/H_2 mixture until the catalyst starts operating in a steady-state mode (i.e. both methanol production and catalyst composition remains stable over the time).

The main changes in Zn K-edge XANES occur in the first 20 minutes after the transient switch from pure hydrogen to CO_2/H_2 mixture as it was already reported in the main text (see Fig. 2). PCA analysis of this dataset is in line with time resolved data shown in Fig. 2b. We have observed oxidative de-alloying of copper-zinc brass and formation of zinc formate reactive intermediate as well as wurtzite like ZnO phase. Methanol MS-response (m/z=31) follows the evolution of zinc formate intermediate. Despite the fact that the main changes in the catalyst composition occur relatively rapidly (during the first 20 minutes after the transient switch), the steady-state operation was not reached at this point of time.

During the next 100 minutes of catalytic reaction over CZA material, we have observed gradual increase of methanol production and simultaneous slow process of copper-zinc brass de-alloying and growth of wurtzite like ZnO phase fraction. Interestingly, the surface zinc formate fraction remains constant during this time. This might indicate that the increase of the catalyst activity in methanol production is due to the de-alloying process and the enrichment of the metallic nanoparticles surface with pure copper, which facilitates hydrogen activation and the subsequent zinc formate hydrogenation to methanol. This was already predicted by theoretical calculations (see Supplementary Table 3) and is in a good agreement with the results of IR study (Fig. 2c) where we observed the low activity of CZA catalyst in the conversion of formate species, when the catalyst is exposed to a highly reducing environment (pure hydrogen) leading to the formation of surface copper-zinc alloy.

Only after 150 minutes following the transient switch to CO_2/H_2 reaction mixture, we observed a steady state regime of the catalyst operation, where both methanol productivity and catalyst composition were unchanged. Supplementary Fig. 16 represents Zn K-edge XANES spectra of CZA catalyst acquired at different time after transient switch from pure hydrogen to CO_2/H_2 reaction mixture indicated with dot lines on Supplementary Fig. 15. As expected from the results of PCA analysis, there is a significant difference in pre-edge feature between the spectrum 1 and spectrum 2, which is indicative of copper-zinc brass de-alloying. However, subsequent spectrum 3 and spectrum 4 collected at the end point of the experiment, represent the same catalyst composition confirming steady-state operation of CZA catalyst after the transient switch.

Supplementary Note 4

Results of chemisorption experiments

In order to investigate metallic copper surface area of CZA catalyst after catalytic methanol synthesis with different activation protocols (see Table 1 for details), oxygen chemisorption experiments by using Micromeritics 3Flex instrument were performed. Sample pre-treatment was done in hydrogen flow at 423 K and ambient pressure in order to minimize reduction of zinc oxide and formation of oxygen vacancies. Isotherms of oxygen adsorption were collected up to 10 mm Hg of pressure. However, it should be noted, that this method is not 100 % selective to titrate metallic surface area of copper and in case of oxygen vacancies formation they will also contribute to total amount of oxygen adsorbed. Therefore, determined values of metallic copper surface area are an upper limit (Supplementary Table 1). Even though, these values correlate well with catalyst activity.

Supplementary Note 5

Additional theoretical information

The calculations revealed that the Gibbs free energy of formation of ZnO layer is becoming more negative with the increased temperature (Supplementary Fig. 17). This means that the higher the temperature during the contact between in situ prepared copper-zinc alloy and CO_2/H_2 mixture is, the higher the degree of copper-zinc alloy oxidation is expected. This agrees well with experimental results.

Theoretical calculations in concert with the experimental observation indicate that surface copper zinc alloy undergo a phase change into copper, zinc oxide and zinc formate; the remaining fraction of reduced zinc is therefore likely captured within the bulk of the copper particle and thus kinetically prevented from oxidation.

As can be seen from the enthalpy of hydrogen chemisorption (Supplementary Table 3), the activation of hydrogen is unlikely over CuZn alloy surface (the exothermic effect of the reaction is only 9.6 $kJ\cdot mol^{-1}$), while pure Cu(111) can activate and split molecular H_2 efficiently (90.7 $kJ\cdot mol^{-1}$).

Supplementary Note 6

Additional transmission electron microscopy observations

Detailed analysis of the lattice space distribution (Supplementary Fig. 20b) suggests the depletion of zinc at the copper-zinc particle surface during carbon dioxide hydrogenation to methanol. It was observed that the interplanar spacing at the edge of copper-zinc particle decreases compared to nanoparticle core. The theoretical interplanar spacing of the {111} planes in copper-zinc alloy is larger compared to that in copper – 0.214 vs. 0.209 nm correspondingly. Zinc de-alloying from the nanoparticle surface results in formation of small (≈ 2nm) zinc oxide nanoparticles with interplanar spacing of 0.25 nm which corresponds to {101} type lattice planes. Furthermore, analysis of EDX spectra extracted from core and shell regions marked on the EDX map (Supplementary Fig. 21), further confirms enrichment of shell with zinc (see ratio between Cu and Zn peaks).

Supplementary Note 7

XANES-PCA study of CuZn-FAU sample

In order to further confirm that CuZn alloy (either in surface or bulk form) is not participating in catalytic mechanism of CO_2 hydrogenation the following sample containing 2.6 wt. % of Cu, 2.4 wt. % of Zn supported on commercial zeolite Y (CBV712, Zeolyst) was investigated by operando XAS during catalytic carbon dioxide hydrogenation.

Supplementary Fig. 23 shows comparison spectra of CZA and CuZn-FAU catalysts during methanol synthesis at 533 K, 15 bar and CO_2:H_2 ratio 1:3. A characteristic pre-edge shoulder at 9659 eV appeared in the Zn K-edge spectrum for CZA sample corresponding to reduced zinc in copper-zinc alloy is not present in CuZn-FAU. XANES-PCA analysis (Supplementary Fig. 24) of time-resolved data acquired during transient switch from CO_2/H_2 mixture to pure hydrogen over CuZn-FAU catalyst revealed the presence of three components: highly dispersed cationic zinc (similar to Zn^{2+} localized in ion-exchange cites of zeolite Y), zinc oxide wurtzite and zinc formate. Zinc formate species formed during catalytic methanol synthesis, undergo hydrogenation to methanol and formation of zinc oxide wurtzite phase.

Thorough analysis (by XANES-PCA and by the absence of any peak at 9659 in difference spectra) reveals the absence reduced zinc in the form of copper-zinc alloy in CuZn/FAU. At the same time the presence of a peak at 9666 eV in the difference spectra (corresponding to the maximum of adsorption of zinc formate), as well as the XANES-PCA analysis, unambiguously provide evidence of participation of surface zinc formate species in catalytic cycle.

Therefore, either presence or absence of copper-zinc alloy is dependent on i) pre-treatment conditions and ii) sample; and is not prerequisite to make catalyst active in carbon dioxide hydrogenation to methanol.

Supplementary Note 8

Additional comments regarding reactive intermediates in catalytic cycle of carbon dioxide hydrogenation

Operando SSITKA-FTIR showed that formates are reactive intermediates to both carbon monoxide and methanol. FTIR alone, however does not reveal the location of the formate species on the bifunctional copper-zinc catalyst. Therefore, operando time-resolved Cu and Zn K-edge XAS was used to probe active sites of the catalyst. Formates species associated with zinc can be hydrogenated to methanol after transient switch from CO_2/H_2 reaction mixture to pure hydrogen (Fig. 2 and Supplementary Figs. 12-13). At the same time, the transient response of produced methanol during this switch exactly follows the trend of zinc-formate species decomposition (Supplementary Fig. 14). Cu K-edge XANES (even for highly dispersed ≈1-2 nm nanoparticles) during a transient switch to hydrogen fails to evidence any changes that can be attributed to presence and hydrogenation (or decomposition) of formate species (Supplementary Figs. 8-10). Therefore, we are claiming that only one observable species which definitely participates in the catalytic cycle of carbon dioxide hydrogenation to methanol over copper-zinc based catalyst is formate associated with zinc. The formation of zinc formate from carbon dioxide and zinc oxide requires reduction of the zinc oxide. The intermediate of this could be oxygen deficient zinc oxide or copper-zinc alloy. It is well documented that deep reduction of zinc oxide yields copper-zinc alloy, which is able to activate carbon dioxide [14,15].

In case of oxidative treatment either in the presence of carbon dioxide (Supplementary Fig. 21, route 2) or oxygen (Supplementary Fig. 25, route 3) copper-zinc catalysts undergo oxidation and form either ZnO-Cu or fully oxidized ZnO-CuO materials.

Supplementary References

1. van den Berg, M. W. E. *et al.* Dynamical changes in the Cu-ZnOx interaction observed in a model methanol synthesis catalyst. *Catal. Letters* **128**, 49–56 (2009).
2. Edwards, A. B., Tildesley, D. J. & Binsted, N. Cumulant expansion analysis of thermal disorder in face centred cubic copper metal by molecular dynamics simulation. *Mol. Phys.* **91**, 357–369 (1997).
3. Clausen, B. S. & Norskov, J. K. Asymmetric pair distribution functions in catalysts. *Top. Catal.* **10**, 221–230 (2000).
4. Jentys, A. Estimation of mean size and shape of small metal particles by EXAFS. *Phys. Chem. Chem. Phys.* **1**, 4059–4063 (1999).
5. Fujita, S. ichiro, Usui, M., Ohara, E. & Takezawa, N. Methanol synthesis from carbon dioxide at atmospheric pressure over Cu/ZnO catalyst. Role of methoxide species formed on ZnO support. *Catal. Letters* **13**, 349–358 (1992).
6. Fujita, S. ichiro, Usui, M., Ito, H. & Takezawa, N. Mechanism of Methanol Synthesis from Carbon Dioxide and from Carbon Monoxide at Atmospheric Pressure over Cu/ZnO. *J. Catal.* **157**, 403–413 (1995).
7. Yang, Y., Mei, D., Peden, C. H. F., Campbell, C. T. & Mims, C. A. Surface-Bound Intermediates in Low-Temperature Methanol Synthesis on Copper: Participants and Spectators. *ACS Catal.* **5**, 7328–7337 (2015).
8. Yang, Y., Mims, C. A., Mei, D. H., Peden, C. H. F. & Campbell, C. T. Mechanistic studies of methanol synthesis over Cu from CO/CO2/H2/H2O mixtures: The source of C in methanol and the role of water. *J. Catal.* **298**, 10–17 (2013).
9. Yang, Y. *et al.* Isotope Effects in Methanol Synthesis and the Reactivity of Copper Formates on a Cu/SiO2 Catalyst. *Catal. Letters* **125**, 201–208 (2008).
10. Yang, Y. *et al.* (Non)formation of methanol by direct hydrogenation of formate on copper catalysts. *J. Phys. Chem. C* **114**, 17205–17211 (2010).
11. Bando, K. K., Sayama, K., Kusama, H., Okabe, K. & Arakawa, H. In-situ FT-IR study on CO2 hydrogenation over Cu catalysts supported on SiO2, Al203, and TiO2. *Appl. Catal. A Gen.* **165**, 391–409 (1997).
12. Kähler, K., Holz, M. C., Rohe, M., Strunk, J. & Muhler, M. Probing the reactivity of ZnO and Au/ZnO nanoparticles by methanol adsorption: A TPD and DRIFTS study. *ChemPhysChem* **11**, 2521–2529 (2010).
13. Fujitani, T. & Nakamura, J. The chemical modification seen in the Cu/ZnO methanol synthesis catalysts. *Appl. Catal. A Gen.* **191**, 111–129 (2000).
14. Behrens, M. *et al.* The active site of methanol synthesis over Cu/ZnO/Al2O3 industrial catalysts. *Science* **336**, 893–897 (2012).

15. Kuld, S. *et al*. Quantifying the promotion of Cu catalysts by ZnO for methanol synthesis. *Science* **352**, 969–974 (2016).
