# Supplementary material for: The unique interplay between copper and zinc during catalytic carbon dioxide hydrogenation to methanol
Source: Nat Commun. 2020 May 15;11:2409. doi: 10.1038/s41467-020-16342-1 (PMC7229192; doi:10.1038/s41467-020-16342-1)
Supplement: Supplementary file 2 — Peer Review File [file 41467_2020_16342_MOESM2_ESM.pdf]

Editorial Note: This manuscript has been previously reviewed at another journal that is not operating a transparent peer review scheme. The manuscript was considered suitable for publication without further review at *Nature Communications*.
